# Supplementary material for: Antibiogram of uropathogens and associated risk factors among asymptomatic female college students in Dessie town, Northeast Ethiopia
Source: PLoS One. 2023 Nov 29;18(11):e0276033. doi: 10.1371/journal.pone.0276033 (PMC10686425; doi:10.1371/journal.pone.0276033)
Supplement: S1 Dataset — (PDF) [file pone.0276033.s001.pdf]

spss data Bre.sav

	ID	Age	AgeDscrptve	college	Residence	Department
1	79	20	15-20	Tropical Co...	Urban	Health Scie...
2	28	19	15-20	Tropical Co...	Rural	Health Scie...
3	47	23	21-25	Tropical Co...	Rural	Health Scie...
4	54	25	21-25	Tropical Co...	Urban	Health Scie...
5	83	18	15-20	Tropical Co...	Urban	Health Scie...
6	95	20	15-20	Tropical Co...	Urban	Health Scie...
7	35	21	21-25	Tropical Co...	Urban	Health Scie...
8	89	18	15-20	Tropical Co...	Urban	Health Scie...
9	62	20	15-20	Tropical Co...	Urban	Health Scie...
10	55	28	26-30	Tropical Co...	Urban	Health Scie...
11	77	18	15-20	Tropical Co...	Urban	Health Scie...
12	84	20	15-20	Tropical Co...	Urban	Health Scie...
13	106	22	21-25	Tropical Co...	Urban	Health Scie...
14	90	19	15-20	Tropical Co...	Urban	Health Scie...
15	70	22	21-25	Tropical Co...	Urban	Health Scie...
16	43	20	15-20	Tropical Co...	Urban	Health Scie...
17	88	20	15-20	Tropical Co...	Urban	Health Scie...
18	12	18	15-20	Tropical Co...	Urban	Health Scie...
19	69	20	15-20	Tropical Co...	Urban	Health Scie...
20	75	19	15-20	Tropical Co...	Urban	Health Scie...
21	86	19	15-20	Tropical Co...	Urban	Health Scie...
22	38	20	15-20	Tropical Co...	Urban	Health Scie...
23	41	21	21-25	Tropical Co...	Urban	Health Scie...
24	29	29	26-30	Tropical Co...	Urban	Health Scie...
25	49	21	21-25	Tropical Co...	Urban	Health Scie...
26	94	19	15-20	Tropical Co...	Urban	Health Scie...
27	44	20	15-20	Tropical Co...	Urban	Health Scie...
28	87	25	21-25	Tropical Co...	Urban	Health Scie...
29	56	25	21-25	Tropical Co...	Urban	Social Scie...
30	33	19	15-20	Tropical Co...	Urban	Social Scie...
31	109	18	15-20	Tropical Co...	Urban	Social Scie...
32	108	22	21-25	Tropical Co...	Urban	Social Scie...
33	43	20	15-20	Tropical Co...	Urban	Social Scie...
34	60	18	15-20	Tropical Co...	Urban	Social Scie...
35	113	19	15-20	Tropical Co...	Urban	Social Scie...

	Batch	MartStatus	Income	Catheter	DM	HIV
1	Second Year	Single	>4000	No	No	No
2	Second Year	Single	>4000	No	No	No
3	Second Year	Single	1001-2000	Yes	No	No
4	Second Year	Single	>4000	Yes	No	No
5	Second Year	Single	3001-4000	No	No	No
6	Second Year	Single	1001-2000	No	No	No
7	Second Year	Single	>4000	No	No	No
8	Second Year	Single	2001-3000	No	No	No
9	Second Year	Single	1001-2000	No	No	No
10	Second Year	Married	>4000	No	No	No
11	Second Year	Single	3001-4000	No	No	No
12	Second Year	Single	>4000	No	No	No
13	Second Year	Married	>4000	No	No	No
14	Second Year	Single	>4000	No	No	No
15	Second Year	Divorced	3001-4000	No	No	No
16	Second Year	Single	≤1000	Yes	No	No
17	Second Year	Single	>4000	No	No	No
18	Second Year	Married	1001-2000	Yes	No	No
19	Second Year	Single	>4000	No	No	Yes
20	Second Year	Single	≤1000	No	No	No
21	Second Year	Single	>4000	No	No	No
22	Second Year	Single	>4000	No	No	No
23	Second Year	Single	>4000	No	No	No
24	Second Year	Single	>4000	No	No	No
25	Second Year	Single	>4000	No	No	Yes
26	Second Year	Single	2001-3000	No	No	No
27	Second Year	Single	>4000	No	No	No
28	Second Year	Single	2001-3000	No	No	No
29	Second Year	Married	>4000	No	No	No
30	Second Year	Single	>4000	No	No	No
31	Second Year	Single	>4000	No	No	No
32	Second Year	Single	2001-3000	No	Yes	No
33	Second Year	Single	>4000	No	No	No
34	Second Year	Single	>4000	No	No	No
35	Second Year	Single	>4000	No	No	No

	STD	Hospitalization	Antibiotics	Contracepive	GentAbnorm	GentWash
1	No	No	No	No	No	During Urin...
2	No	No	No	No	No	During Urin...
3	No	No	No	No	No	During Urin...
4	No	No	No	IUD	Yes	During Urin...
5	No	No	No	Injection	No	During Urin...
6	No	No	No	No	No	During Urin...
7	No	No	No	Implant	No	During Urin...
8	No	No	No	Injection	No	During Urin...
9	No	No	No	Injection	No	During Urin...
10	No	No	No	Pill	Yes	During Urin...
11	No	No	No	No	No	During Urin...
12	No	No	No	Implant	No	During Urin...
13	No	No	No	Pill	No	During Urin...
14	No	No	No	No	No	During Urin...
15	No	No	No	Pill	No	During Urin...
16	No	No	Yes	Pill	No	others
17	No	No	No	No	No	During Urin...
18	No	No	No	No	No	during devi...
19	No	No	No	No	No	During Urin...
20	No	No	No	No	Yes	during devi...
21	No	No	No	No	No	others
22	No	No	No	Condom	No	during devi...
23	No	No	No	No	No	During Urin...
24	No	No	No	IUD	No	During Urin...
25	No	No	No	No	No	During Urin...
26	No	No	No	No	No	During Urin...
27	No	No	No	No	Yes	During Urin...
28	No	No	No	No	No	During Urin...
29	No	No	No	No	No	During Urin...
30	No	No	No	No	No	During Urin...
31	No	No	No	No	No	During Urin...
32	No	No	No	No	No	During Urin...
33	No	No	No	No	No	During Urin...
34	No	No	No	No	No	During Urin...
35	No	No	No	No	No	During Urin...

	SexFrequency	SU	BactSpp	FungSpp	CL	E
1	NO	Negative	.	.	.	.
2	NO	Negative	.	.	.	.
3	NO	Negative	.	.	.	.
4	NO	Negative	.	.	.	.
5	NO	Postive	.	Candidia al...	.	.
6	<3 times	Negative	.	.	.	.
7	<3 times	Postive	Providencia...	.	.	.
8	NO	Postive	E.coli	.	.	.
9	<3 times	Postive	.	Candidia gl...	.	.
10	<3 times	Negative	.	.	.	.
11	<3 times	Negative	.	.	.	.
12	<3 times	Postive	Citrobacter ...	.	.	.
13	<3 times	Negative	.	.	.	.
14	NO	Negative	.	.	.	.
15	NO	Negative	.	.	.	.
16	<3 times	Postive	Citrobacter ...	.	.	.
17	<3 times	Negative	.	.	.	.
18	<3 times	Negative	.	.	.	.
19	>=3 times	Negative	.	.	.	.
20	NO	Negative	.	.	.	.
21	>=3 times	Postive	E.coli	.	.	.
22	<3 times	Postive	.	Candidia tr...	.	.
23	NO	Negative	.	.	.	.
24	NO	Negative	.	.	.	.
25	NO	Negative	.	.	.	.
26	NO	Negative	.	.	.	.
27	NO	Negative	.	.	.	.
28	NO	Negative	.	.	.	.
29	<3 times	Negative	.	.	.	.
30	NO	Negative	.	.	.	.
31	NO	Negative	.	.	.	.
32	NO	Negative	.	.	.	.
33	NO	Negative	.	.	.	.
34	NO	Negative	.	.	.	.
35	NO	Negative	.	.	.	.

spss data Bre.sav

	PEN	CAF	CIP	TTC	SXT	F
1	.	.	.	.	.	.
2	.	.	.	.	.	.
3	.	.	.	.	.	.
4	.	.	.	.	.	.
5	.	.	.	.	.	.
6	.	.	.	.	.	.
7	.	.	Sensetive	.	Resistant	Sensetive
8	.	.	Intermidate	Resistant	Intermidiate	Sensetive
9	.	.	.	.	.	.
10	.	.	.	.	.	.
11	.	.	.	.	.	.
12	.	.	Sensetive	Resistant	Sensetive	Resistant
13	.	.	.	.	.	.
14	.	.	.	.	.	.
15	.	.	.	.	.	.
16	.	.	Sensetive	Sensetive	Sensetive	Sensetive
17	.	.	.	.	.	.
18	.	.	.	.	.	.
19	.	.	.	.	.	.
20	.	.	.	.	.	.
21	.	.	Resistant	Sensetive	Sensetive	Sensetive
22	.	.	.	.	.	.
23	.	.	.	.	.	.
24	.	.	.	.	.	.
25	.	.	.	.	.	.
26	.	.	.	.	.	.
27	.	.	.	.	.	.
28	.	.	.	.	.	.
29	.	.	.	.	.	.
30	.	.	.	.	.	.
31	.	.	.	.	.	.
32	.	.	.	.	.	.
33	.	.	.	.	.	.
34	.	.	.	.	.	.
35	.	.	.	.	.	.

spss data Bre.sav

	NOR	CRO	AMC	CTX	CAZ	AMP
1	.	.	.	.	.	.
2	.	.	.	.	.	.
3	.	.	.	.	.	.
4	.	.	.	.	.	.
5	.	.	.	.	.	.
6	.	.	.	.	.	.
7	Resistant	Intermidiate	Resistant	Intermidiate	Sensetive	Sensetive
8	Sensetive	Resistant	Resistant	Resistant	Resistant	Intermidiate
9	.	.	.	.	.	.
10	.	.	.	.	.	.
11	.	.	.	.	.	.
12	Sensetive	Sensetive	Resistant	Sensetive	Sensetive	Resistant
13	.	.	.	.	.	.
14	.	.	.	.	.	.
15	.	.	.	.	.	.
16	Sensetive	Sensetive	Resistant	Sensetive	Sensetive	Sensetive
17	.	.	.	.	.	.
18	.	.	.	.	.	.
19	.	.	.	.	.	.
20	.	.	.	.	.	.
21	Resistant	Sensetive	Resistant	Sensetive	Sensetive	Resistant
22	.	.	.	.	.	.
23	.	.	.	.	.	.
24	.	.	.	.	.	.
25	.	.	.	.	.	.
26	.	.	.	.	.	.
27	.	.	.	.	.	.
28	.	.	.	.	.	.
29	.	.	.	.	.	.
30	.	.	.	.	.	.
31	.	.	.	.	.	.
32	.	.	.	.	.	.
33	.	.	.	.	.	.
34	.	.	.	.	.	.
35	.	.	.	.	.	.

spss data Bre.sav

	AMK	GEN	ResPattern	MDR	GENTTWASH
1	.	.	.	.	Afterurination
2	.	.	.	.	Afterurination
3	.	.	.	.	Afterurination
4	.	.	.	.	Afterurination
5	.	.	.	.	Afterdefication
6	.	.	.	.	Afterdefication
7	Sensetive	.	>=R5	Yes	Afterurination
8	Resistant	Sensetive	>=R5	Yes	Afterdefication
9	.	.	.	.	Afterdefication
10	.	.	.	.	Afterurination
11	.	.	.	.	Afterdefication
12	Sensetive	Intermidate	>=R5	Yes	Afterurination
13	.	.	.	.	Afterurination
14	.	.	.	.	Afterurination
15	.	.	.	.	Afterurination
16	Sensetive	Sensetive	R1	No	Afterurination
17	.	.	.	.	Afterurination
18	.	.	.	.	Afterdefication
19	.	.	.	.	Afterurination
20	.	.	.	.	Afterdefication
21	Sensetive	Sensetive	R4	No	Afterurination
22	.	.	.	.	Afterurination
23	.	.	.	.	Afterurination
24	.	.	.	.	Afterurination
25	.	.	.	.	Afterurination
26	.	.	.	.	Afterurination
27	.	.	.	.	Afterurination
28	.	.	.	.	Afterurination
29	.	.	.	.	Afterurination
30	.	.	.	.	Afterurination
31	.	.	.	.	Afterurination
32	.	.	.	.	Everymorning
33	.	.	.	.	Afterurination
34	.	.	.	.	Afterurination
35	.	.	.	.	Afterurination

spss data Bre.sav

	ID	Age	AgeDscrptve	college	Residence	Department
36	15	20	15-20	Tropical Co...	Urban	Social Scie...
37	32	20	15-20	Tropical Co...	Urban	Social Scie...
38	26	19	15-20	Tropical Co...	Urban	Social Scie...
39	25	25	21-25	Tropical Co...	Urban	Social Scie...
40	85	19	15-20	Tropical Co...	Urban	Social Scie...
41	59	23	21-25	Tropical Co...	Urban	Social Scie...
42	111	24	21-25	Tropical Co...	Rural	Social Scie...
43	7	18	15-20	Tropical Co...	Urban	Natural Sci...
44	103	20	15-20	Tropical Co...	Urban	Natural Sci...
45	16	19	15-20	Tropical Co...	Urban	Natural Sci...
46	46	20	15-20	Dessie Hea...	Urban	Health Scie...
47	107	20	15-20	Dessie Hea...	Urban	Health Scie...
48	31	38	>30	Dessie Hea...	Urban	Health Scie...
49	34	20	15-20	Dessie Hea...	Urban	Health Scie...
50	64	20	15-20	Dessie Hea...	Urban	Health Scie...
51	44	21	21-25	Dessie Hea...	Urban	Health Scie...
52	42	23	21-25	Dessie Hea...	Urban	Health Scie...
53	104	20	15-20	Dessie Hea...	Urban	Health Scie...
54	54	24	21-25	Dessie Hea...	Urban	Health Scie...
55	41	31	>30	Dessie Hea...	Urban	Health Scie...
56	45	22	21-25	Dessie Hea...	Urban	Health Scie...
57	82	20	15-20	Dessie Hea...	Urban	Health Scie...
58	61	20	15-20	Dessie Hea...	Urban	Health Scie...
59	51	20	15-20	Dessie Hea...	Urban	Health Scie...
60	63	20	15-20	Dessie Hea...	Urban	Health Scie...
61	37	20	15-20	Dessie Hea...	Urban	Health Scie...
62	43	20	15-20	Dessie Hea...	Rural	Health Scie...
63	52	20	15-20	Dessie Hea...	Urban	Health Scie...
64	24	23	21-25	Dessie Hea...	Urban	Health Scie...
65	66	19	15-20	Dessie Hea...	Urban	Health Scie...
66	15	22	21-25	Dessie Hea...	Urban	Health Scie...
67	30	20	15-20	Dessie Hea...	Urban	Health Scie...
68	63	19	15-20	Dessie Hea...	Urban	Health Scie...
69	.	.	.	.	.	.
70	18	20	15-20	Dessie Hea...	Urban	Health Scie...

	Batch	MartStatus	Income	Catheter	DM	HIV
36	Second Year	Single	>4000	No	No	No
37	Second Year	Single	≤1000	No	No	No
38	Second Year	Single	1001-2000	Yes	No	No
39	Second Year	Single	≤1000	No	No	No
40	Second Year	Single	≤1000	No	No	No
41	Second Year	Single	>4000	No	No	No
42	Second Year	Divorced	>4000	No	No	No
43	Second Year	Single	>4000	No	No	No
44	Second Year	Single	≤1000	No	No	No
45	Second Year	Single	>4000	No	No	No
46	Second Year	Single	3001-4000	No	No	No
47	Second Year	Single	3001-4000	No	No	No
48	Second Year	Married	>4000	No	No	No
49	Second Year	Married	3001-4000	No	No	No
50	Second Year	Single	>4000	No	No	No
51	Second Year	Single	3001-4000	No	No	No
52	Second Year	Single	>4000	No	No	No
53	Second Year	Single	>4000	No	No	No
54	Second Year	Single	1001-2000	No	No	No
55	Second Year	Single	≤1000	No	No	No
56	Second Year	Single	1001-2000	No	No	No
57	Second Year	Single	>4000	No	No	No
58	Second Year	Single	>4000	No	No	No
59	Second Year	Single	>4000	No	No	No
60	Second Year	Single	≤1000	No	No	No
61	Second Year	Single	1001-2000	No	No	No
62	Second Year	Single	≤1000	No	No	No
63	Second Year	Single	3001-4000	No	No	No
64	Second Year	Married	3001-4000	No	No	No
65	Second Year	Single	>4000	No	No	No
66	Second Year	Divorced	2001-3000	No	No	No
67	Second Year	Married	>4000	No	No	No
68	Second Year	Married	>4000	No	No	No
69	.	.	.	.	.	.
70	Second Year	Single	>4000	No	No	No

	STD	Hospitalization	Antibiotics	Contracepive	GentAbnorm	GentWash
36	No	No	No	No	No	During Urin...
37	No	No	No	No	No	during devi...
38	No	Yes	Yes	No	No	During Urin...
39	No	Yes	Yes	No	No	During Urin...
40	No	No	No	No	No	During Urin...
41	No	No	No	No	No	During Urin...
42	No	No	Yes	Implant	Yes	During Urin...
43	No	Yes	Yes	No	No	every morni...
44	No	No	No	No	No	others
45	No	No	No	No	No	During Urin...
46	No	No	No	No	No	During Urin...
47	No	No	No	No	No	During Urin...
48	No	No	No	IUD	No	during devi...
49	No	No	No	No	No	every morni...
50	No	No	No	No	No	During Urin...
51	No	Yes	Yes	No	No	others
52	No	No	No	Condom	No	During Urin...
53	No	No	No	No	No	During Urin...
54	No	No	No	No	No	during devi...
55	No	No	No	IUD	No	During Urin...
56	No	No	No	No	No	During Urin...
57	No	No	No	No	No	During Urin...
58	No	No	No	No	No	during devi...
59	No	No	No	No	No	During Urin...
60	No	No	No	No	No	During Urin...
61	No	No	No	No	No	during devi...
62	No	No	No	No	No	others
63	No	No	No	No	No	during devi...
64	No	No	No	Implant	No	During Urin...
65	No	No	No	Implant	No	others
66	No	No	No	Implant	No	During Urin...
67	No	No	No	No	No	others
68	No	Yes	Yes	Pill	No	every morni...
69	.	.	.	.	.	.
70	No	No	No	Injection	No	during devi...

	SexFrequency	SU	BactSpp	FungSpp	CL	E
36	<3 times	Negative	.	.	.	.
37	NO	Negative	.	.	.	.
38	>=3 times	Postive	.	Candidia tr...	.	.
39	NO	Negative	.	.	.	.
40	NO	Negative	.	.	.	.
41	NO	Negative	.	.	.	.
42	NO	Negative	.	.	.	.
43	NO	Postive	Providencia...	Candidia tr...	.	.
44	NO	Negative	.	.	.	.
45	NO	Negative	.	.	.	.
46	NO	Negative	.	.	.	.
47	NO	Negative	.	.	.	.
48	<3 times	Negative	.	.	.	.
49	<3 times	Negative	.	.	.	.
50	NO	Negative	.	.	.	.
51	NO	Postive	.	Candidia kr...	.	.
52	<3 times	Negative	.	.	.	.
53	NO	Negative	.	.	.	.
54	NO	Postive	Klebsella o...	.	.	.
55	NO	Negative	.	.	.	.
56	NO	Negative	.	.	.	.
57	NO	Negative	.	.	.	.
58	NO	Negative	.	.	.	.
59	NO	Negative	.	.	.	.
60	NO	Negative	.	.	.	.
61	NO	Negative	.	.	.	.
62	<3 times	Postive	Klebsella r...	.	.	.
63	NO	Negative	.	.	.	.
64	NO	Negative	.	.	.	.
65	NO	Negative	.	.	.	.
66	NO	Negative	.	.	.	.
67	NO	Negative	.	.	.	.
68	NO	Postive	.	Candidia al...	.	.
69	.	.	.	Candidia kr...	.	.
70	NO	Negative	.	.	.	.

	PEN	CAF	CIP	TTC	SXT	F
36	.	.	.	.	.	.
37	.	.	.	.	.	.
38	.	.	.	.	.	.
39	.	.	.	.	.	.
40	.	.	.	.	.	.
41	.	.	.	.	.	.
42	.	.	.	.	.	.
43	.	.	Sensetive	.	Resistant	Sensetive
44	.	.	.	.	.	.
45	.	.	.	.	.	.
46	.	.	.	.	.	.
47	.	.	.	.	.	.
48	.	.	.	.	.	.
49	.	.	.	.	.	.
50	.	.	.	.	.	.
51	.	.	.	.	.	.
52	.	.	.	.	.	.
53	.	.	.	.	.	.
54	.	.	Sensetive	Sensetive	Sensetive	Sensetive
55	.	.	.	.	.	.
56	.	.	.	.	.	.
57	.	.	.	.	.	.
58	.	.	.	.	.	.
59	.	.	.	.	.	.
60	.	.	.	.	.	.
61	.	.	.	.	.	.
62	.	.	Sensetive	Resistant	Resistant	Sensetive
63	.	.	.	.	.	.
64	.	.	.	.	.	.
65	.	.	.	.	.	.
66	.	.	.	.	.	.
67	.	.	.	.	.	.
68	.	.	.	.	.	.
69	.	.	.	.	.	.
70	.	.	.	.	.	.

	NOR	CRO	AMC	CTX	CAZ	AMP
36	.	.	.	.	.	.
37	.	.	.	.	.	.
38	.	.	.	.	.	.
39	.	.	.	.	.	.
40	.	.	.	.	.	.
41	.	.	.	.	.	.
42	.	.	.	.	.	.
43	Sensetive	Resistant	Resistant	Resistant	Intermidate	Sensetive
44	.	.	.	.	.	.
45	.	.	.	.	.	.
46	.	.	.	.	.	.
47	.	.	.	.	.	.
48	.	.	.	.	.	.
49	.	.	.	.	.	.
50	.	.	.	.	.	.
51	.	.	.	.	.	.
52	.	.	.	.	.	.
53	.	.	.	.	.	.
54	Sensetive	Sensetive	Resistant	Sensetive	Intermidate	.
55	.	.	.	.	.	.
56	.	.	.	.	.	.
57	.	.	.	.	.	.
58	.	.	.	.	.	.
59	.	.	.	.	.	.
60	.	.	.	.	.	.
61	.	.	.	.	.	.
62	Sensetive	Resistant	Resistant	Resistant	Resistant	.
63	.	.	.	.	.	.
64	.	.	.	.	.	.
65	.	.	.	.	.	.
66	.	.	.	.	.	.
67	.	.	.	.	.	.
68	.	.	.	.	.	.
69	.	.	.	.	.	.
70	.	.	.	.	.	.

spss data Bre.sav

	AMK	GEN	ResPattern	MDR	GENTTWASH
36	.	.	.	.	Afterurination
37	.	.	.	.	Afterdefication
38	.	.	.	.	Afterurination
39	.	.	.	.	Everymorning
40	.	.	.	.	Afterurination
41	.	.	.	.	Afterurination
42	.	.	.	.	Afterurination
43	Intermidiate	.	>=R5	Yes	Afterurination
44	.	.	.	.	Others
45	.	.	.	.	Afterurination
46	.	.	.	.	Afterurination
47	.	.	.	.	Everymorning
48	.	.	.	.	Afterdefication
49	.	.	.	.	Everymorning
50	.	.	.	.	Afterurination
51	.	.	.	.	Afterurination
52	.	.	.	.	Afterurination
53	.	.	.	.	Afterurination
54	Sensetive	Resistant	R3	No	Afterdefication
55	.	.	.	.	Afterurination
56	.	.	.	.	Afterurination
57	.	.	.	.	Afterurination
58	.	.	.	.	Afterurination
59	.	.	.	.	Afterurination
60	.	.	.	.	Afterurination
61	.	.	.	.	Afterdefication
62	Sensetive	Sensetive	>=R5	Yes	Afterdefication
63	.	.	.	.	Afterdefication
64	.	.	.	.	Afterurination
65	.	.	.	.	Others
66	.	.	.	.	Afterurination
67	.	.	.	.	Others
68	.	.	.	.	Afterdefication
69	.	.	.	.	.
70	.	.	.	.	Afterdefication

	ID	Age	AgeDscrptve	college	Residence	Department
71	69	20	15-20	Dessie Hea...	Urban	Health Scie...
72	6	21	21-25	Dessie Hea...	Urban	Health Scie...
73	60	22	21-25	Dessie Hea...	Urban	Health Scie...
74	21	21	21-25	Dessie Hea...	Urban	Health Scie...
75	49	19	15-20	Dessie Hea...	Urban	Health Scie...
76	46	23	21-25	Dessie Hea...	Urban	Health Scie...
77	69	18	15-20	Dessie Hea...	Urban	Health Scie...
78	91	22	21-25	Dessie Hea...	Urban	Health Scie...
79	40	24	21-25	Dessie Hea...	Urban	Health Scie...
80	68	22	21-25	Dessie Hea...	Urban	Health Scie...
81	65	22	21-25	Dessie Hea...	Urban	Health Scie...
82	79	20	15-20	Dessie Hea...	Urban	Health Scie...
83	83	23	21-25	Dessie Hea...	Urban	Health Scie...
84	73	20	15-20	Dessie Hea...	Urban	Health Scie...
85	39	29	26-30	Dessie Hea...	Urban	Health Scie...
86	38	19	15-20	Dessie Hea...	Urban	Health Scie...
87	106	20	15-20	Dessie Hea...	Rural	Health Scie...
88	37	19	15-20	Dessie Hea...	Urban	Health Scie...
89	55	20	15-20	Dessie Hea...	Urban	Health Scie...
90	87	19	15-20	Dessie Hea...	Rural	Health Scie...
91	81	21	21-25	Dessie Hea...	Urban	Health Scie...
92	102	19	15-20	Dessie Hea...	Urban	Health Scie...
93	65	20	15-20	Dessie Hea...	Rural	Health Scie...
94	103	19	15-20	Dessie Hea...	Urban	Health Scie...
95	95	21	21-25	Dessie Hea...	Urban	Health Scie...
96	85	20	15-20	Dessie Hea...	Rural	Health Scie...
97	100	23	21-25	Dessie Hea...	Rural	Health Scie...
98	73	22	21-25	Dessie Hea...	Urban	Health Scie...
99	67	20	15-20	Dessie Hea...	Rural	Health Scie...
100	63	32	>30	Dessie Hea...	Urban	Health Scie...
101	96	23	21-25	Dessie Hea...	Urban	Health Scie...
102	68	23	21-25	Dessie Hea...	Urban	Health Scie...
103	83	20	15-20	Dessie Hea...	Rural	Health Scie...
104	64	21	21-25	Dessie Hea...	Rural	Health Scie...
105	76	20	15-20	Dessie Hea...	Urban	Health Scie...

	Batch	MartStatus	Income	Catheter	DM	HIV
71	Second Year	Single	1001-2000	No	No	No
72	Second Year	Single	1001-2000	No	No	No
73	Second Year	Single	1001-2000	No	No	No
74	Second Year	Divorced	≤1000	No	No	No
75	Second Year	Single	2001-3000	No	No	No
76	Second Year	Single	≤1000	No	No	No
77	Second Year	Single	1001-2000	No	No	No
78	Second Year	Single	1001-2000	No	No	No
79	Second Year	Single	1001-2000	No	No	No
80	Second Year	Single	>4000	No	No	No
81	Second Year	Divorced	>4000	No	No	No
82	Second Year	Single	>4000	No	No	No
83	Second Year	Married	1001-2000	No	No	No
84	Second Year	Married	1001-2000	No	No	No
85	Second Year	Married	>4000	No	No	No
86	Second Year	Married	>4000	No	No	No
87	Second Year	Single	>4000	No	No	No
88	Second Year	Single	3001-4000	No	No	No
89	Second Year	Single	>4000	No	No	No
90	Second Year	Single	3001-4000	No	No	No
91	Second Year	Single	>4000	Yes	No	No
92	Second Year	Single	3001-4000	No	No	No
93	Second Year	Single	3001-4000	No	No	No
94	Second Year	Single	1001-2000	No	No	No
95	Second Year	Divorced	≤1000	No	No	No
96	Second Year	Single	1001-2000	No	No	No
97	Second Year	Single	≤1000	No	No	No
98	Second Year	Single	1001-2000	No	No	No
99	Second Year	Single	2001-3000	No	No	No
100	Second Year	Single	>4000	No	No	No
101	Second Year	Single	>4000	No	No	No
102	Second Year	Married	3001-4000	Yes	No	No
103	Second Year	Single	2001-3000	No	No	No
104	Second Year	Single	>4000	No	No	No
105	Second Year	Single	1001-2000	No	No	No

	STD	Hospitalization	Antibiotics	Contracepive	GentAbnorm	GentWash
71	No	No	No	Implant	No	during devi...
72	No	No	No	No	No	during devi...
73	No	No	No	No	No	During Urin...
74	No	No	No	Injection	No	During Urin...
75	No	No	No	No	No	During Urin...
76	No	No	No	Injection	No	every morni...
77	No	No	No	No	No	During Urin...
78	No	No	No	No	No	During Urin...
79	No	No	No	No	No	During Urin...
80	No	No	No	No	No	during devi...
81	No	No	No	Implant	No	During Urin...
82	No	No	No	No	No	During Urin...
83	No	No	No	Injection	No	During Urin...
84	No	No	Yes	Implant	No	During Urin...
85	No	No	Yes	No	No	During Urin...
86	No	No	No	Implant	Yes	During Urin...
87	No	No	No	No	No	During Urin...
88	No	No	Yes	No	No	during devi...
89	No	No	No	No	No	during devi...
90	No	No	No	No	No	During Urin...
91	No	No	No	Implant	No	every morni...
92	No	No	No	No	No	During Urin...
93	No	No	No	No	No	during devi...
94	No	No	No	Implant	No	during devi...
95	No	Yes	Yes	Condom	No	during devi...
96	No	No	No	No	No	during devi...
97	No	No	No	No	No	during devi...
98	No	No	No	No	No	during devi...
99	No	No	No	No	No	during devi...
100	No	No	No	IUD	No	during devi...
101	No	No	No	No	No	During Urin...
102	No	Yes	Yes	Implant	No	during devi...
103	No	No	No	No	No	During Urin...
104	No	No	No	Implant	No	during devi...
105	No	No	No	No	No	During Urin...

	SexFrequency	SU	BactSpp	FungSpp	CL	E
71	<3 times	Negative	.	.	.	.
72	NO	Negative	.	.	.	.
73	NO	Negative	.	.	.	.
74	<3 times	Negative	.	.	.	.
75	NO	Negative	.	.	.	.
76	<3 times	Negative	.	.	.	.
77	NO	Negative	.	.	.	.
78	>=3 times	Postive	E.coli	.	.	.
79	NO	Negative	.	.	.	.
80	NO	Negative	.	.	.	.
81	NO	Negative	.	.	.	.
82	NO	Negative	.	.	.	.
83	NO	Negative	.	.	.	.
84	<3 times	Negative	.	.	.	.
85	NO	Negative	.	.	.	.
86	<3 times	Negative	.	.	.	.
87	NO	Negative	.	.	.	.
88	NO	Negative	.	.	.	.
89	NO	Negative	.	.	.	.
90	NO	Negative	.	.	.	.
91	<3 times	Postive	S. saproph...	.	Sensetive	.
92	NO	Negative	.	.	.	.
93	NO	Postive	S. saproph...	.	Resistant	.
94	NO	Negative	.	.	.	.
95	NO	Negative	.	.	.	.
96	NO	Postive	serratia	.	.	.
97	NO	Negative	.	.	.	.
98	NO	Postive	.	Candidia gl...	.	.
99	NO	Postive	S. saproph...	.	Intermideate	.
100	NO	Negative	.	.	.	.
101	NO	Negative	.	.	.	.
102	<3 times	Postive	klebsella p...	.	.	.
103	NO	Negative	.	.	.	.
104	NO	Postive	.	Candidia kr...	.	.
105	NO	Negative	.	.	.	.

spss data Bre.sav

	PEN	CAF	CIP	TTC	SXT	F
71	.	.	.	.	.	.
72	.	.	.	.	.	.
73	.	.	.	.	.	.
74	.	.	.	.	.	.
75	.	.	.	.	.	.
76	.	.	.	.	.	.
77	.	.	.	.	.	.
78	.	.	Sensetive	Sensetive	Resistant	Sensetive
79	.	.	.	.	.	.
80	.	.	.	.	.	.
81	.	.	.	.	.	.
82	.	.	.	.	.	.
83	.	.	.	.	.	.
84	.	.	.	.	.	.
85	.	.	.	.	.	.
86	.	.	.	.	.	.
87	.	.	.	.	.	.
88	.	.	.	.	.	.
89	.	.	.	.	.	.
90	.	.	.	.	.	.
91	Resistant	Resistant	Sensetive	Intermidate	Resistant	Sensetive
92	.	.	.	.	.	.
93	Resistant	Sensetive	Sensetive	Sensetive	Sensetive	Sensetive
94	.	.	.	.	.	.
95	.	.	.	.	.	.
96	.	.	Sensetive	Sensetive	Intermidiate	Sensetive
97	.	.	.	.	.	.
98	.	.	.	.	.	.
99	Resistant	Sensetive	Sensetive	Intermidate	Resistant	Sensetive
100	.	.	.	.	.	.
101	.	.	.	.	.	.
102	.	.	Sensetive	Sensetive	Sensetive	Intermidiate
103	.	.	.	.	.	.
104	.	.	.	.	.	.
105	.	.	.	.	.	.

	NOR	CRO	AMC	CTX	CAZ	AMP
71	.	.	.	.	.	.
72	.	.	.	.	.	.
73	.	.	.	.	.	.
74	.	.	.	.	.	.
75	.	.	.	.	.	.
76	.	.	.	.	.	.
77	.	.	.	.	.	.
78	Sensetive	Intermidiate	Intermidiate	Sensetive	Resistant	Resistant
79	.	.	.	.	.	.
80	.	.	.	.	.	.
81	.	.	.	.	.	.
82	.	.	.	.	.	.
83	.	.	.	.	.	.
84	.	.	.	.	.	.
85	.	.	.	.	.	.
86	.	.	.	.	.	.
87	.	.	.	.	.	.
88	.	.	.	.	.	.
89	.	.	.	.	.	.
90	.	.	.	.	.	.
91	Sensetive	.	.	.	.	.
92	.	.	.	.	.	.
93	Sensetive	.	.	.	.	.
94	.	.	.	.	.	.
95	.	.	.	.	.	.
96	Sensetive	Resistant	.	Resistant	Sensetive	.
97	.	.	.	.	.	.
98	.	.	.	.	.	.
99	Sensetive	.	.	.	.	.
100	.	.	.	.	.	.
101	.	.	.	.	.	.
102	Intermidiate	Resistant	Resistant	Resistant	Sensetive	.
103	.	.	.	.	.	.
104	.	.	.	.	.	.
105	.	.	.	.	.	.

spss data Bre.sav

	AMK	GEN	ResPattern	MDR	GENTTWASH
71	.	.	.	.	Afterdefication
72	.	.	.	.	Afterurination
73	.	.	.	.	Everymorning
74	.	.	.	.	Everymorning
75	.	.	.	.	Afterurination
76	.	.	.	.	Everymorning
77	.	.	.	.	Afterurination
78	Sensetive	Intermidate	>=R5	Yes	Afterurination
79	.	.	.	.	Afterdefication
80	.	.	.	.	Afterdefication
81	.	.	.	.	Afterurination
82	.	.	.	.	Afterurination
83	.	.	.	.	Afterurination
84	.	.	.	.	Afterurination
85	.	.	.	.	Afterurination
86	.	.	.	.	Afterurination
87	.	.	.	.	Afterurination
88	.	.	.	.	Afterurination
89	.	.	.	.	Afterurination
90	.	.	.	.	Afterurination
91	.	.	R4	Yes	Afterurination
92	.	.	.	.	Afterurination
93	.	.	R2	No	Afterdefication
94	.	.	.	.	Afterdefication
95	.	.	.	.	Afterurination
96	Sensetive	Sensetive	R3	No	Afterdefication
97	.	.	.	.	Afterdefication
98	.	.	.	.	Afterdefication
99	.	.	R4	Yes	Afterurination
100	.	.	.	.	Afterurination
101	.	.	.	.	Afterurination
102	Sensetive	Sensetive	>=R5	Yes	Afterurination
103	.	.	.	.	Afterdefication
104	.	.	.	.	Afterdefication
105	.	.	.	.	Afterdefication

	ID	Age	AgeDscrptve	college	Residence	Department
106	9	22	21-25	Dessie Hea...	Urban	Health Scie...
107	79	22	21-25	Dessie Hea...	Urban	Health Scie...
108	75	25	21-25	Dessie Hea...	Urban	Health Scie...
109	51	21	21-25	Dessie Hea...	Urban	Health Scie...
110	98	22	21-25	Dessie Hea...	Urban	Health Scie...
111	30	19	15-20	Dessie Hea...	Urban	Health Scie...
112	52	23	21-25	Dessie Hea...	Urban	Health Scie...
113	82	20	15-20	Dessie Hea...	Urban	Health Scie...
114	36	20	15-20	Dessie Hea...	Urban	Health Scie...
115	62	20	15-20	Tropical Co...	Rural	Health Scie...
116	88	21	21-25	Tropical Co...	Urban	Health Scie...
117	47	20	15-20	Tropical Co...	Urban	Health Scie...
118	28	22	21-25	Tropical Co...	Urban	Health Scie...
119	24	20	15-20	Tropical Co...	Rural	Health Scie...
120	46	20	15-20	Tropical Co...	Urban	Health Scie...
121	33	21	21-25	Tropical Co...	Urban	Health Scie...
122	25	23	21-25	Tropical Co...	Urban	Health Scie...
123	10	22	21-25	Tropical Co...	Urban	Health Scie...
124	48	20	15-20	Tropical Co...	Urban	Health Scie...
125	50	21	21-25	Tropical Co...	Urban	Health Scie...
126	26	22	21-25	Tropical Co...	Urban	Health Scie...
127	39	20	15-20	Tropical Co...	Urban	Health Scie...
128	20	21	21-25	Tropical Co...	Urban	Health Scie...
129	11	21	21-25	Tropical Co...	Urban	Health Scie...
130	23	20	15-20	Tropical Co...	Urban	Health Scie...
131	18	20	15-20	Tropical Co...	Urban	Health Scie...
132	70	24	21-25	Tropical Co...	Urban	Health Scie...
133	106	21	21-25	Tropical Co...	Urban	Health Scie...
134	52	21	21-25	Tropical Co...	Urban	Health Scie...
135	22	19	15-20	Tropical Co...	Urban	Social Scie...
136	24	21	21-25	Tropical Co...	Urban	Social Scie...
137	32	21	21-25	Tropical Co...	Urban	Social Scie...
138	47	20	15-20	Tropical Co...	Urban	Social Scie...
139	40	20	15-20	Tropical Co...	Urban	Social Scie...
140	60	21	21-25	Tropical Co...	Urban	Social Scie...

	Batch	MartStatus	Income	Catheter	DM	HIV
106	Second Year	Married	1001-2000	No	No	No
107	Second Year	Single	1001-2000	No	No	No
108	Second Year	Married	1001-2000	No	No	No
109	Second Year	Divorced	1001-2000	No	No	No
110	Second Year	Married	≤1000	No	No	No
111	Second Year	Single	>4000	No	No	No
112	Second Year	Single	>4000	No	No	No
113	Second Year	Single	>4000	No	No	No
114	Second Year	Married	>4000	No	No	No
115	Third Year	Single	>4000	No	Yes	No
116	Third Year	Single	>4000	No	No	No
117	Third Year	Single	>4000	No	No	No
118	Third Year	Single	1001-2000	No	No	No
119	Third Year	Single	2001-3000	No	No	No
120	Third Year	Single	1001-2000	No	No	No
121	Third Year	Single	2001-3000	No	No	No
122	Third Year	Married	2001-3000	No	No	No
123	Third Year	Married	>4000	Yes	No	No
124	Third Year	Single	≤1000	No	No	No
125	Third Year	Married	>4000	No	No	No
126	Third Year	Married	1001-2000	No	No	No
127	Third Year	Single	>4000	No	No	No
128	Third Year	Single	>4000	No	No	No
129	Third Year	Single	≤1000	No	No	No
130	Third Year	Single	>4000	No	No	No
131	Third Year	Single	≤1000	No	No	No
132	Third Year	Single	>4000	No	No	No
133	Third Year	Single	1001-2000	No	No	No
134	Third Year	Single	3001-4000	No	No	No
135	Third Year	Single	>4000	No	No	No
136	Third Year	Single	>4000	No	No	No
137	Third Year	Married	>4000	No	No	No
138	Third Year	Single	3001-4000	No	No	No
139	Third Year	Single	1001-2000	No	No	No
140	Third Year	Single	>4000	No	No	No

	STD	Hospitalization	Antibiotics	Contracepive	GentAbnorm	GentWash
106	No	No	No	Pill	No	During Urin...
107	No	Yes	Yes	No	No	during devi...
108	No	No	No	Implant	No	during devi...
109	No	No	No	Implant	No	during devi...
110	No	No	No	No	Yes	During Urin...
111	No	Yes	Yes	No	No	During Urin...
112	No	No	No	No	No	During Urin...
113	No	No	No	Implant	Yes	during devi...
114	No	No	Yes	No	No	During Urin...
115	No	No	No	No	No	During Urin...
116	No	No	No	No	No	During Urin...
117	No	Yes	Yes	No	No	During Urin...
118	No	No	No	Implant	No	during devi...
119	No	No	No	Condom	No	during devi...
120	No	No	No	No	No	during devi...
121	No	No	No	No	No	during devi...
122	No	No	No	Injection	Yes	During Urin...
123	No	No	No	Implant	No	During Urin...
124	No	No	No	Condom	No	during devi...
125	No	No	No	Pill	No	During Urin...
126	No	No	No	Injection	No	During Urin...
127	No	No	No	No	No	during devi...
128	No	No	No	No	No	during devi...
129	No	No	No	Implant	Yes	during devi...
130	No	No	No	Injection	No	during devi...
131	No	No	No	Injection	No	during devi...
132	No	No	No	Injection	No	during devi...
133	No	No	No	No	No	During Urin...
134	No	Yes	Yes	No	No	During Urin...
135	No	No	No	No	No	During Urin...
136	No	No	No	No	Yes	During Urin...
137	No	No	No	Injection	No	During Urin...
138	No	No	No	No	No	During Urin...
139	No	No	No	No	No	During Urin...
140	No	No	No	No	No	During Urin...

	SexFrequency	SU	BactSpp	FungSpp	CL	E
106	NO	Negative	.	.	.	.
107	NO	Negative	.	.	.	.
108	NO	Negative	.	.	.	.
109	NO	Negative	.	.	.	.
110	<3 times	Negative	.	.	.	.
111	NO	Negative	.	.	.	.
112	NO	Negative	.	.	.	.
113	NO	Postive	S. saproph...	Candidia tr...	Sensetive	.
114	<3 times	Negative	.	.	.	.
115	NO	Negative	.	.	.	.
116	NO	Negative	.	.	.	.
117	NO	Negative	.	.	.	.
118	<3 times	Postive	.	Candidia kr...	.	.
119	NO	Postive	S. saproph...	.	Resistant	.
120	>=3 times	Postive	Klebsella r...	.	.	.
121	>=3 times	Postive	Acinetobac...	.	.	.
122	NO	Negative	.	.	.	.
123	>=3 times	Postive	Klebsella o...	.	.	.
124	NO	Postive	S. saproph...	.	Intermideate	.
125	NO	Postive	.	Candidia gl...	.	.
126	<3 times	Negative	.	.	.	.
127	NO	Negative	.	.	.	.
128	NO	Negative	.	.	.	.
129	NO	Postive	Klebsella o...	.	.	.
130	NO	Negative	.	.	.	.
131	NO	Postive	.	Candidia tr...	.	.
132	>=3 times	Postive	.	Candidia tr...	.	.
133	NO	Negative	.	.	.	.
134	NO	Negative	.	.	.	.
135	NO	Negative	.	.	.	.
136	NO	Negative	.	.	.	.
137	<3 times	Negative	.	.	.	.
138	NO	Negative	.	.	.	.
139	NO	Negative	.	.	.	.
140	NO	Negative	.	.	.	.

	PEN	CAF	CIP	TTC	SXT	F
106	.	.	.	.	.	.
107	.	.	.	.	.	.
108	.	.	.	.	.	.
109	.	.	.	.	.	.
110	.	.	.	.	.	.
111	.	.	.	.	.	.
112	.	.	.	.	.	.
113	Resistant	Sensetive	Resistant	Sensetive	Resistant	Sensetive
114	.	.	.	.	.	.
115	.	.	.	.	.	.
116	.	.	.	.	.	.
117	.	.	.	.	.	.
118	.	.	.	.	.	.
119	Resistant	Sensetive	Resistant	Sensetive	Resistant	Sensetive
120	.	.	Sensetive	Resistant	Resistant	Sensetive
121	.	.	Sensetive	Resistant	Resistant	Sensetive
122	.	.	.	.	.	.
123	.	.	Sensetive	Intermidate	Resistant	Resistant
124	Resistant	Sensetive	Sensetive	Resistant	Intermidiate	Sensetive
125	.	.	.	.	.	.
126	.	.	.	.	.	.
127	.	.	.	.	.	.
128	.	.	.	.	.	.
129	.	.	Sensetive	Sensetive	Resistant	Resistant
130	.	.	.	.	.	.
131	.	.	.	.	.	.
132	.	.	.	.	.	.
133	.	.	.	.	.	.
134	.	.	.	.	.	.
135	.	.	.	.	.	.
136	.	.	.	.	.	.
137	.	.	.	.	.	.
138	.	.	.	.	.	.
139	.	.	.	.	.	.
140	.	.	.	.	.	.

	NOR	CRO	AMC	CTX	CAZ	AMP
106	.	.	.	.	.	.
107	.	.	.	.	.	.
108	.	.	.	.	.	.
109	.	.	.	.	.	.
110	.	.	.	.	.	.
111	.	.	.	.	.	.
112	.	.	.	.	.	.
113	Resistant	.	.	.	.	.
114	.	.	.	.	.	.
115	.	.	.	.	.	.
116	.	.	.	.	.	.
117	.	.	.	.	.	.
118	.	.	.	.	.	.
119	Sensetive	.	.	.	.	.
120	Sensetive	Resistant	Resistant	Resistant	Resistant	Sensetive
121	Sensetive	Resistant	Resistant	Resistant	Resistant	.
122	.	.	.	.	.	.
123	Sensetive	Resistant	Resistant	Resistant	Resistant	.
124	Resistant	.	.	.	.	.
125	.	.	.	.	.	.
126	.	.	.	.	.	.
127	.	.	.	.	.	.
128	.	.	.	.	.	.
129	Sensetive	Sensetive	Resistant	Sensetive	Sensetive	.
130	.	.	.	.	.	.
131	.	.	.	.	.	.
132	.	.	.	.	.	.
133	.	.	.	.	.	.
134	.	.	.	.	.	.
135	.	.	.	.	.	.
136	.	.	.	.	.	.
137	.	.	.	.	.	.
138	.	.	.	.	.	.
139	.	.	.	.	.	.
140	.	.	.	.	.	.

	AMK	GEN	ResPattern	MDR	GENTTWASH
106	.	.	.	.	After urination
107	.	.	.	.	After defication
108	.	.	.	.	After defication
109	.	.	.	.	After defication
110	.	.	.	.	After urination
111	.	.	.	.	After urination
112	.	.	.	.	After urination
113	.	.	R4	Yes	After urination
114	.	.	.	.	After defication
115	.	.	.	.	After urination
116	.	.	.	.	After urination
117	.	.	.	.	After urination
118	.	.	.	.	After urination
119	.	.	R4	Yes	After urination
120	Sensetive	Sensetive	>=R5	Yes	After defication
121	Resistant	Sensetive	>=R5	Yes	After urination
122	.	.	.	.	After defication
123	Sensetive	Sensetive	>=R5	Yes	After urination
124	.	.	R4	Yes	After defication
125	.	.	.	.	After urination
126	.	.	.	.	After urination
127	.	.	.	.	After defication
128	.	.	.	.	Others
129	Resistant	Sensetive	R4	Yes	After urination
130	.	.	.	.	After defication
131	.	.	.	.	After defication
132	.	.	.	.	After defication
133	.	.	.	.	After defication
134	.	.	.	.	After urination
135	.	.	.	.	After urination
136	.	.	.	.	After urination
137	.	.	.	.	After urination
138	.	.	.	.	After defication
139	.	.	.	.	After defication
140	.	.	.	.	After urination

	ID	Age	AgeDscrptve	college	Residence	Department
141	25	20	15-20	Tropical Co...	Urban	Social Scie...
142	13	21	21-25	Tropical Co...	Urban	Social Scie...
143	33	21	21-25	Tropical Co...	Urban	Social Scie...
144	10	21	21-25	Tropical Co...	Urban	Natural Sci...
145	16	20	15-20	Tropical Co...	Urban	Natural Sci...
146	48	31	>30	Tropical Co...	Urban	Health Scie...
147	49	23	21-25	Tropical Co...	Urban	Health Scie...
148	45	26	26-30	Tropical Co...	Urban	Health Scie...
149	43	24	21-25	Tropical Co...	Urban	Health Scie...
150	53	26	26-30	Tropical Co...	Urban	Health Scie...
151	27	23	21-25	Tropical Co...	Urban	Health Scie...
152	50	23	21-25	Tropical Co...	Urban	Health Scie...
153	55	26	26-30	Tropical Co...	Urban	Health Scie...
154	34	22	21-25	Tropical Co...	Urban	Health Scie...
155	51	22	21-25	Tropical Co...	Urban	Health Scie...
156	11	23	21-25	Tropical Co...	Urban	Health Scie...
157	44	23	21-25	Tropical Co...	Urban	Health Scie...
158	54	26	26-30	Tropical Co...	Urban	Health Scie...
159	31	23	21-25	Tropical Co...	Urban	Health Scie...
160	58	22	21-25	Tropical Co...	Urban	Health Scie...
161	41	24	21-25	Tropical Co...	Urban	Health Scie...
162	35	26	26-30	Tropical Co...	Urban	Health Scie...
163	28	23	21-25	Tropical Co...	Urban	Health Scie...
164	3	23	21-25	Tropical Co...	Urban	Health Scie...
165	59	26	26-30	Tropical Co...	Urban	Health Scie...
166	2	22	21-25	Tropical Co...	Urban	Health Scie...
167	29	22	21-25	Tropical Co...	Urban	Health Scie...
168	139	23	21-25	Tropical Co...	Urban	Health Scie...
169	161	22	21-25	Tropical Co...	Urban	Health Scie...
170	168	23	21-25	Tropical Co...	Urban	Health Scie...
171	129	22	21-25	Tropical Co...	Urban	Health Scie...
172	165	26	26-30	Tropical Co...	Urban	Health Scie...
173	151	25	21-25	Tropical Co...	Urban	Health Scie...
174	147	23	21-25	Tropical Co...	Urban	Health Scie...
175	180	31	>30	Tropical Co...	Urban	Health Scie...

	Batch	MartStatus	Income	Catheter	DM	HIV
141	Third Year	Married	>4000	No	No	No
142	Third Year	Single	>4000	No	No	No
143	Third Year	Single	>4000	No	No	No
144	Third Year	Single	>4000	No	No	No
145	Third Year	Single	>4000	No	No	No
146	Fourth Year	Single	>4000	No	No	No
147	Fourth Year	Single	>4000	No	No	No
148	Fourth Year	Single	>4000	No	No	No
149	Fourth Year	Single	>4000	No	No	No
150	Fourth Year	Single	>4000	No	No	No
151	Fourth Year	Single	>4000	No	No	No
152	Fourth Year	Single	2001-3000	No	No	No
153	Fourth Year	Single	>4000	No	No	No
154	Fourth Year	Single	≤1000	No	No	No
155	Fourth Year	Divorced	>4000	No	No	No
156	Fourth Year	Single	>4000	No	No	No
157	Fourth Year	Single	>4000	No	No	No
158	Fourth Year	Married	>4000	No	No	No
159	Fourth Year	Single	>4000	No	No	No
160	Fourth Year	Single	>4000	No	No	No
161	Fourth Year	Single	>4000	No	No	No
162	Fourth Year	Single	>4000	No	No	No
163	Fourth Year	Single	>4000	No	No	No
164	Fourth Year	Single	2001-3000	No	No	No
165	Fourth Year	Single	3001-4000	No	No	No
166	Fourth Year	Single	>4000	No	No	No
167	Fourth Year	Single	3001-4000	No	No	No
168	Fourth Year	Single	3001-4000	No	No	No
169	Fourth Year	Single	>4000	No	No	No
170	Fourth Year	Single	>4000	No	Yes	No
171	Fourth Year	Single	3001-4000	No	No	No
172	Fourth Year	Single	>4000	No	No	No
173	Fourth Year	Single	>4000	No	No	No
174	Fourth Year	Single	>4000	No	No	No
175	Fourth Year	Single	>4000	No	No	No

spss data Bre.sav

	STD	Hospitalization	Antibiotics	Contracepive	GentAbnorm	GentWash
141	No	No	Yes	Pill	No	During Urin...
142	No	No	No	Condom	No	During Urin...
143	No	No	No	Condom	No	during devi...
144	No	No	No	No	No	during devi...
145	No	No	No	No	No	During Urin...
146	No	No	No	IUD	No	During Urin...
147	No	No	No	No	No	During Urin...
148	No	No	No	No	No	During Urin...
149	No	No	No	Condom	No	During Urin...
150	No	No	No	No	Yes	During Urin...
151	No	No	Yes	Injection	Yes	During Urin...
152	No	No	No	Injection	No	during devi...
153	No	No	No	No	No	During Urin...
154	No	No	No	No	No	During Urin...
155	No	No	No	Pill	No	During Urin...
156	No	No	Yes	No	Yes	during devi...
157	No	No	No	No	No	During Urin...
158	No	No	No	Injection	No	During Urin...
159	No	No	No	No	No	During Urin...
160	No	No	No	No	No	During Urin...
161	No	No	No	Condom	Yes	During Urin...
162	No	No	No	No	Yes	During Urin...
163	No	No	Yes	No	No	During Urin...
164	No	No	No	Condom	No	during devi...
165	No	No	No	Injection	No	during devi...
166	No	No	No	No	No	During Urin...
167	No	No	No	No	No	During Urin...
168	No	No	No	Injection	No	during devi...
169	No	No	No	Injection	No	during devi...
170	No	No	No	No	No	During Urin...
171	No	No	No	Injection	No	during devi...
172	Yes	No	No	Pill	Yes	others
173	No	No	No	No	No	During Urin...
174	No	No	Yes	Pill	No	During Urin...
175	No	No	No	Implant	No	During Urin...

	SexFrequency	SU	BactSpp	FungSpp	CL	E
141	<3 times	Postive	S. saproph...	.	Sensetive	.
142	<3 times	Negative	.	.	.	.
143	<3 times	Postive	S. saproph...	.	Sensetive	.
144	NO	Negative	.	.	.	.
145	NO	Negative	.	.	.	.
146	NO	Negative	.	.	.	.
147	NO	Negative	.	.	.	.
148	NO	Negative	.	.	.	.
149	NO	Negative	.	.	.	.
150	NO	Negative	.	.	.	.
151	NO	Postive	S. saproph...	.	Resistant	.
152	NO	Postive	S. saproph...	Candidia tr...	Intermideate	.
153	NO	Negative	.	.	.	.
154	NO	Postive	.	Candidia tr...	.	.
155	<3 times	Negative	.	.	.	.
156	NO	Postive	E.coli	.	.	.
157	NO	Negative	.	.	.	.
158	<3 times	Negative	.	.	.	.
159	NO	Negative	.	.	.	.
160	NO	Negative	.	.	.	.
161	<3 times	Negative	.	.	.	.
162	NO	Negative	.	.	.	.
163	NO	Negative	.	.	.	.
164	NO	Negative	.	.	.	.
165	NO	Postive	S. saproph...	Candidia kr...	Sensetive	.
166	<3 times	Negative	.	.	.	.
167	NO	Negative	.	.	.	.
168	NO	Postive	.	Candidia al...	.	.
169	NO	Postive	.	Candidia kr...	.	.
170	NO	Negative	.	.	.	.
171	NO	Postive	S.aureus	.	Sensetive	.
172	<3 times	Negative	.	.	.	.
173	<3 times	Negative	.	.	.	.
174	NO	Negative	.	.	.	.
175	NO	Negative	.	.	.	.

spss data Bre.sav

	PEN	CAF	CIP	TTC	SXT	F
141	Resistant	Sensetive	Resistant	Sensetive	Sensetive	Intermidiate
142	.	.	.	.	.	.
143	Resistant	Resistant	Intermidate	Intermidate	Resistant	Sensetive
144	.	.	.	.	.	.
145	.	.	.	.	.	.
146	.	.	.	.	.	.
147	.	.	.	.	.	.
148	.	.	.	.	.	.
149	.	.	.	.	.	.
150	.	.	.	.	.	.
151	Resistant	Resistant	Sensetive	Resistant	Sensetive	Sensetive
152	Resistant	Sensetive	Sensetive	Resistant	Resistant	Sensetive
153	.	.	.	.	.	.
154	.	.	.	.	.	.
155	.	.	.	.	.	.
156	.	.	Sensetive	Resistant	Resistant	Sensetive
157	.	.	.	.	.	.
158	.	.	.	.	.	.
159	.	.	.	.	.	.
160	.	.	.	.	.	.
161	.	.	.	.	.	.
162	.	.	.	.	.	.
163	.	.	.	.	.	.
164	.	.	.	.	.	.
165	Sensetive	Sensetive	Resistant	Intermidate	Resistant	Intermidiate
166	.	.	.	.	.	.
167	.	.	.	.	.	.
168	.	.	.	.	.	.
169	.	.	.	.	.	.
170	.	.	.	.	.	.
171	Resistant	Sensetive	Sensetive	Intermidate	Sensetive	Sensetive
172	.	.	.	.	.	.
173	.	.	.	.	.	.
174	.	.	.	.	.	.
175	.	.	.	.	.	.

	NOR	CRO	AMC	CTX	CAZ	AMP
141	Sensetive	.	.	.	.	.
142	.	.	.	.	.	.
143	Resistant	.	.	.	.	.
144	.	.	.	.	.	.
145	.	.	.	.	.	.
146	.	.	.	.	.	.
147	.	.	.	.	.	.
148	.	.	.	.	.	.
149	.	.	.	.	.	.
150	.	.	.	.	.	.
151	Resistant	.	.	.	.	.
152	Sensetive	.	.	.	.	.
153	.	.	.	.	.	.
154	.	.	.	.	.	.
155	.	.	.	.	.	.
156	Sensetive	Intermidiate	Intermidiate	Intermidate	Sensetive	Resistant
157	.	.	.	.	.	.
158	.	.	.	.	.	.
159	.	.	.	.	.	.
160	.	.	.	.	.	.
161	.	.	.	.	.	.
162	.	.	.	.	.	.
163	.	.	.	.	.	.
164	.	.	.	.	.	.
165	Sensetive	.	.	.	.	.
166	.	.	.	.	.	.
167	.	.	.	.	.	.
168	.	.	.	.	.	.
169	.	.	.	.	.	.
170	.	.	.	.	.	.
171	Intermidiate	.	.	.	.	.
172	.	.	.	.	.	.
173	.	.	.	.	.	.
174	.	.	.	.	.	.
175	.	.	.	.	.	.

	AMK	GEN	ResPattern	MDR	GENTTWASH
141	.	.	R3	Yes	Afterurination
142	.	.	.	.	Afterurination
143	.	.	>=R5	Yes	Afterurination
144	.	.	.	.	Afterdefication
145	.	.	.	.	Afterurination
146	.	.	.	.	Afterurination
147	.	.	.	.	Afterdefication
148	.	.	.	.	Afterurination
149	.	.	.	.	Afterurination
150	.	.	.	.	Afterurination
151	.	.	>=R5	Yes	Afterurination
152	.	.	R4	Yes	Afterdefication
153	.	.	.	.	Afterurination
154	.	.	.	.	Afterurination
155	.	.	.	.	Afterurination
156	Intermidiate	Sensetive	>=R5	Yes	Afterdefication
157	.	.	.	.	Afterurination
158	.	.	.	.	Afterurination
159	.	.	.	.	Afterurination
160	.	.	.	.	Afterurination
161	.	.	.	.	Afterurination
162	.	.	.	.	Afterurination
163	.	.	.	.	Afterurination
164	.	.	.	.	Afterdefication
165	.	.	R4	Yes	Afterurination
166	.	.	.	.	Afterurination
167	.	.	.	.	Afterurination
168	.	.	.	.	Afterurination
169	.	.	.	.	Afterurination
170	.	.	.	.	Afterurination
171	.	.	R3	Yes	Afterdefication
172	.	.	.	.	Everymorning
173	.	.	.	.	Afterurination
174	.	.	.	.	Afterurination
175	.	.	.	.	Afterurination

	ID	Age	AgeDscrptve	college	Residence	Department
176	144	22	21-25	Tropical Co...	Urban	Health Scie...
177	142	22	21-25	Tropical Co...	Urban	Health Scie...
178	127	32	>30	Tropical Co...	Urban	Health Scie...
179	170	23	21-25	Tropical Co...	Urban	Health Scie...
180	162	24	21-25	Tropical Co...	Urban	Health Scie...
181	160	22	21-25	Tropical Co...	Urban	Health Scie...
182	159	23	21-25	Tropical Co...	Urban	Health Scie...
183	132	31	>30	Tropical Co...	Urban	Health Scie...
184	81	22	21-25	Tropical Co...	Urban	Health Scie...
185	71	23	21-25	Tropical Co...	Urban	Health Scie...
186	73	24	21-25	Tropical Co...	Urban	Health Scie...
187	63	22	21-25	Tropical Co...	Urban	Health Scie...
188	85	31	>30	Tropical Co...	Urban	Health Scie...
189	108	23	21-25	Tropical Co...	Urban	Health Scie...
190	104	22	21-25	Tropical Co...	Urban	Health Scie...
191	61	30	26-30	Tropical Co...	Urban	Health Scie...
192	115	24	21-25	Tropical Co...	Urban	Health Scie...
193	109	28	26-30	Tropical Co...	Urban	Health Scie...
194	111	25	21-25	Tropical Co...	Urban	Health Scie...
195	78	31	>30	Tropical Co...	Urban	Health Scie...
196	68	30	26-30	Tropical Co...	Urban	Health Scie...
197	103	23	21-25	Tropical Co...	Urban	Health Scie...
198	181	33	>30	Tropical Co...	Urban	Health Scie...
199	65	25	21-25	Tropical Co...	Urban	Health Scie...
200	93	31	>30	Tropical Co...	Urban	Health Scie...
201	83	24	21-25	Tropical Co...	Urban	Health Scie...
202	112	21	21-25	Alkan Healt...	Urban	Health Scie...
203	81	19	15-20	Alkan Healt...	Urban	Health Scie...
204	89	20	15-20	Alkan Healt...	Urban	Health Scie...
205	84	19	15-20	Alkan Healt...	Urban	Health Scie...
206	87	21	21-25	Alkan Healt...	Urban	Health Scie...
207	117	21	21-25	Alkan Healt...	Urban	Health Scie...
208	94	21	21-25	Alkan Healt...	Urban	Health Scie...
209	83	22	21-25	Alkan Healt...	Urban	Health Scie...
210	55	24	21-25	Alkan Healt...	Urban	Health Scie...

	Batch	MartStatus	Income	Catheter	DM	HIV
176	Fourth Year	Single	3001-4000	No	No	Yes
177	Fourth Year	Single	>4000	No	No	No
178	Fourth Year	Single	>4000	No	No	No
179	Fourth Year	Married	>4000	No	Yes	No
180	Fourth Year	Single	1001-2000	No	No	No
181	Fourth Year	Single	>4000	Yes	No	No
182	Fourth Year	Single	>4000	No	No	No
183	Fourth Year	Single	>4000	No	No	No
184	Fourth Year	Single	1001-2000	No	No	No
185	Fourth Year	Single	3001-4000	Yes	Yes	No
186	Fourth Year	Single	>4000	No	No	No
187	Fourth Year	Single	>4000	No	No	No
188	Fourth Year	Married	>4000	No	Yes	No
189	Fourth Year	Single	>4000	No	No	No
190	Fourth Year	Single	>4000	No	No	No
191	Fourth Year	Married	>4000	No	No	No
192	Fourth Year	Single	>4000	No	No	No
193	Fourth Year	Married	>4000	No	No	No
194	Fourth Year	Married	>4000	Yes	No	No
195	Fourth Year	Single	3001-4000	No	No	No
196	Fourth Year	Single	>4000	No	No	No
197	Fourth Year	Single	>4000	No	No	No
198	Fourth Year	Married	>4000	No	No	No
199	Fourth Year	Married	>4000	No	No	No
200	Fourth Year	Married	>4000	No	No	No
201	Fourth Year	Single	>4000	No	No	No
202	Second Year	Single	>4000	No	No	Yes
203	Second Year	Single	>4000	No	No	No
204	Second Year	Single	>4000	No	No	No
205	Second Year	Single	>4000	No	No	No
206	Second Year	Single	3001-4000	No	No	No
207	Second Year	Single	>4000	No	No	No
208	Second Year	Single	3001-4000	No	No	No
209	Second Year	Single	>4000	No	No	No
210	Second Year	Single	>4000	No	No	No

	STD	Hospitalization	Antibiotics	Contracepive	GentAbnorm	GentWash
176	No	No	No	No	No	every morni...
177	No	No	No	Injection	Yes	During Urin...
178	No	No	Yes	Injection	No	During Urin...
179	No	No	Yes	Pill	No	During Urin...
180	No	No	No	No	No	During Urin...
181	Yes	No	No	No	Yes	others
182	No	No	No	Pill	No	During Urin...
183	No	Yes	Yes	No	No	During Urin...
184	No	No	No	Pill	No	During Urin...
185	No	No	No	Pill	No	During Urin...
186	No	No	No	No	No	during devi...
187	No	No	No	No	No	During Urin...
188	No	No	No	Pill	No	during devi...
189	No	No	No	No	No	during devi...
190	No	No	No	No	No	During Urin...
191	No	No	No	Pill	No	During Urin...
192	No	No	No	No	No	During Urin...
193	No	No	Yes	No	Yes	During Urin...
194	No	No	No	Implant	No	During Urin...
195	No	Yes	Yes	No	No	during devi...
196	No	No	No	Pill	No	During Urin...
197	No	No	No	No	No	During Urin...
198	No	No	No	No	No	During Urin...
199	No	No	No	No	No	during devi...
200	No	No	No	No	No	During Urin...
201	No	No	Yes	IUD	Yes	During Urin...
202	No	No	No	No	No	During Urin...
203	No	No	No	No	No	every morni...
204	No	No	No	Condom	No	During Urin...
205	No	No	No	No	No	During Urin...
206	No	No	No	No	No	During Urin...
207	No	No	No	No	No	During Urin...
208	No	No	No	Pill	No	During Urin...
209	No	No	No	No	No	During Urin...
210	No	No	No	Implant	No	During Urin...

	SexFrequency	SU	BactSpp	FungSpp	CL	E
176	NO	Negative	.	.	.	.
177	NO	Postive	S.epidermidis	Candidia tr...	Sensetive	.
178	NO	Postive	S. saproph...	.	Sensetive	.
179	NO	Negative	.	.	.	.
180	NO	Negative	.	.	.	.
181	NO	Negative	.	.	.	.
182	NO	Negative	.	.	.	.
183	NO	Negative	.	.	.	.
184	NO	Negative	.	.	.	.
185	NO	Negative	.	.	.	.
186	NO	Postive	.	Candidia tr...	.	.
187	NO	Negative	.	.	.	.
188	<3 times	Postive	S. saproph...	.	Resistant	.
189	NO	Postive	.	Candidia tr...	.	.
190	NO	Postive	S. saproph...	.	Sensetive	.
191	<3 times	Negative	.	.	.	.
192	NO	Negative	.	.	.	.
193	<3 times	Negative	.	.	.	.
194	<3 times	Negative	.	.	.	.
195	NO	Postive	.	Candidia tr...	.	.
196	>=3 times	Postive	.	Candidia tr...	.	.
197	NO	Negative	.	.	.	.
198	NO	Negative	.	.	.	.
199	NO	Postive	S. saproph...	.	Sensetive	.
200	NO	Negative	.	.	.	.
201	NO	Negative	.	.	.	.
202	NO	Negative	.	.	.	.
203	NO	Negative	.	.	.	.
204	NO	Negative	.	.	.	.
205	NO	Negative	.	.	.	.
206	NO	Negative	.	.	.	.
207	NO	Negative	.	.	.	.
208	<3 times	Negative	.	.	.	.
209	NO	Negative	.	.	.	.
210	<3 times	Postive	Citrobacter	.	.	.

...	PEN	CAF	CIP	TTC	SXT	F
176	.	.	.	.	.	.
177	Resistant	Sensetive	Sensetive	Resistant	Intermidiate	Sensetive
178	Resistant	Sensetive	Sensetive	Sensetive	Resistant	Sensetive
179	.	.	.	.	.	.
180	.	.	.	.	.	.
181	.	.	.	.	.	.
182	.	.	.	.	.	.
183	.	.	.	.	.	.
184	.	.	.	.	.	.
185	.	.	.	.	.	.
186	.	.	.	.	.	.
187	.	.	.	.	.	.
188	Resistant	Resistant	Intermidate	Resistant	Resistant	Sensetive
189	.	.	.	.	.	.
190	Resistant	Resistant	Intermidate	Resistant	Resistant	Sensetive
191	.	.	.	.	.	.
192	.	.	.	.	.	.
193	.	.	.	.	.	.
194	.	.	.	.	.	.
195	.	.	.	.	.	.
196	.	.	.	.	.	.
197	.	.	.	.	.	.
198	.	.	.	.	.	.
199	Resistant	Resistant	Sensetive	Resistant	Resistant	Sensetive
200	.	.	.	.	.	.
201	.	.	.	.	.	.
202	.	.	.	.	.	.
203	.	.	.	.	.	.
204	.	.	.	.	.	.
205	.	.	.	.	.	.
206	.	.	.	.	.	.
207	.	.	.	.	.	.
208	.	.	.	.	.	.
209	.	.	.	.	.	.
210	.	.	Intermidate	Resistant	Resistant	Sensetive

...	NOR	CRO	AMC	CTX	CAZ	AMP
176	.	.	.	.	.	.
177	Sensetive	.	.	.	.	.
178	Sensetive	.	.	.	.	.
179	.	.	.	.	.	.
180	.	.	.	.	.	.
181	.	.	.	.	.	.
182	.	.	.	.	.	.
183	.	.	.	.	.	.
184	.	.	.	.	.	.
185	.	.	.	.	.	.
186	.	.	.	.	.	.
187	.	.	.	.	.	.
188	Sensetive	.	.	.	.	.
189	.	.	.	.	.	.
190	Sensetive	.	.	.	.	.
191	.	.	.	.	.	.
192	.	.	.	.	.	.
193	.	.	.	.	.	.
194	.	.	.	.	.	.
195	.	.	.	.	.	.
196	.	.	.	.	.	.
197	.	.	.	.	.	.
198	.	.	.	.	.	.
199	Sensetive	.	.	.	.	.
200	.	.	.	.	.	.
201	.	.	.	.	.	.
202	.	.	.	.	.	.
203	.	.	.	.	.	.
204	.	.	.	.	.	.
205	.	.	.	.	.	.
206	.	.	.	.	.	.
207	.	.	.	.	.	.
208	.	.	.	.	.	.
209	.	.	.	.	.	.
210	Sensetive	Sensetive	Resistant	Sensetive	Intermidate	Resistant

	AMK	GEN	ResPattern	MDR	GENTTWASH
176	.	.	.	.	Everymorning
177	.	.	R3	Yes	Afterurination
178	.	.	R2	No	Afterurination
179	.	.	.	.	Afterurination
180	.	.	.	.	Afterurination
181	.	.	.	.	Others
182	.	.	.	.	Afterurination
183	.	.	.	.	Afterurination
184	.	.	.	.	Afterurination
185	.	.	.	.	Afterdefication
186	.	.	.	.	Afterurination
187	.	.	.	.	Afterurination
188	.	.	>=R5	Yes	Afterurination
189	.	.	.	.	Afterurination
190	.	.	>=R5	Yes	Afterurination
191	.	.	.	.	Afterdefication
192	.	.	.	.	Afterurination
193	.	.	.	.	Afterurination
194	.	.	.	.	Afterdefication
195	.	.	.	.	Afterurination
196	.	.	.	.	Afterurination
197	.	.	.	.	Afterurination
198	.	.	.	.	Afterurination
199	.	.	R4	Yes	Afterurination
200	.	.	.	.	Afterurination
201	.	.	.	.	Afterdefication
202	.	.	.	.	Afterurination
203	.	.	.	.	Afterurination
204	.	.	.	.	Afterurination
205	.	.	.	.	Afterurination
206	.	.	.	.	Afterurination
207	.	.	.	.	Afterurination
208	.	.	.	.	Afterdefication
209	.	.	.	.	Afterurination
210	Sensetive	Intermidate	>=R5	Yes	Afterurination

	ID	Age	AgeDscrptve	college	Residence	Department
211	92	32	>30	Alkan Healt...	Urban	Health Scie...
212	104	23	21-25	Alkan Healt...	Urban	Health Scie...
213	110	21	21-25	Alkan Healt...	Urban	Health Scie...
214	79	22	21-25	Alkan Healt...	Urban	Health Scie...
215	101	23	21-25	Alkan Healt...	Urban	Health Scie...
216	10	22	21-25	Alkan Healt...	Rural	Health Scie...
217	24	21	21-25	Alkan Healt...	Urban	Health Scie...
218	108	20	15-20	Alkan Healt...	Urban	Health Scie...
219	87	20	15-20	Alkan Healt...	Urban	Health Scie...
220	89	19	15-20	Alkan Healt...	Urban	Health Scie...
221	54	21	21-25	Alkan Healt...	Urban	Health Scie...
222	60	20	15-20	Alkan Healt...	Urban	Health Scie...
223	106	21	21-25	Alkan Healt...	Urban	Health Scie...
224	28	18	15-20	Alkan Healt...	Urban	Health Scie...
225	29	21	21-25	Alkan Healt...	Urban	Health Scie...
226	23	20	15-20	Alkan Healt...	Urban	Health Scie...
227	4	24	21-25	Alkan Healt...	Urban	Social Scie...
228	30	20	15-20	Alkan Healt...	Urban	Social Scie...
229	48	21	21-25	Alkan Healt...	Urban	Social Scie...
230	14	21	21-25	Alkan Healt...	Urban	Social Scie...
231	20	20	15-20	Alkan Healt...	Urban	Social Scie...
232	22	21	21-25	Alkan Healt...	Urban	Social Scie...
233	26	21	21-25	Alkan Healt...	Urban	Social Scie...
234	21	20	15-20	Alkan Healt...	Urban	Social Scie...
235	34	22	21-25	Alkan Healt...	Urban	Social Scie...
236	45	22	21-25	Alkan Healt...	Urban	Social Scie...
237	25	20	15-20	Alkan Healt...	Urban	Social Scie...
238	31	19	15-20	Alkan Healt...	Rural	Social Scie...
239	42	23	21-25	Alkan Healt...	Rural	Social Scie...
240	44	25	21-25	Alkan Healt...	Urban	Social Scie...
241	3	18	15-20	Alkan Healt...	Urban	Social Scie...
242	56	20	15-20	Alkan Healt...	Urban	Social Scie...
243	12	21	21-25	Alkan Healt...	Urban	Health Scie...
244	19	18	15-20	Alkan Healt...	Urban	Health Scie...
245	28	20	15-20	Alkan Healt...	Urban	Health Scie...

...	Batch	MartStatus	Income	Catheter	DM	HIV
211	Second Year	Single	>4000	No	No	No
212	Second Year	Single	>4000	No	No	No
213	Second Year	Single	>4000	No	No	No
214	Second Year	Single	2001-3000	No	No	No
215	Second Year	Married	>4000	No	No	No
216	Second Year	Single	>4000	No	No	No
217	Second Year	Single	3001-4000	No	No	No
218	Second Year	Single	≤1000	No	No	No
219	Second Year	Single	1001-2000	No	No	No
220	Second Year	Married	2001-3000	No	No	No
221	Second Year	Married	>4000	No	No	No
222	Second Year	Married	>4000	No	No	No
223	Second Year	Single	>4000	No	No	No
224	Second Year	Single	>4000	No	No	No
225	Second Year	Single	1001-2000	No	No	No
226	Second Year	Single	1001-2000	No	No	No
227	Second Year	Single	3001-4000	No	No	No
228	Second Year	Single	>4000	No	No	No
229	Second Year	Single	>4000	No	No	No
230	Second Year	Single	>4000	No	No	No
231	Second Year	Single	3001-4000	No	No	No
232	Second Year	Single	>4000	No	No	No
233	Second Year	Single	>4000	No	No	No
234	Second Year	Single	>4000	No	No	No
235	Second Year	Single	>4000	No	No	No
236	Second Year	Single	>4000	No	No	No
237	Second Year	Single	>4000	No	No	No
238	Second Year	Single	>4000	No	No	No
239	Second Year	Single	1001-2000	No	No	No
240	Second Year	Single	>4000	Yes	No	No
241	Second Year	Single	3001-4000	No	No	No
242	Second Year	Single	1001-2000	No	No	No
243	Third Year	Single	>4000	No	No	No
244	Third Year	Single	2001-3000	No	No	No
245	Third Year	Single	1001-2000	No	No	No

	STD	Hospitalization	Antibiotics	Contracepive	GentAbnorm	GentWash
211	No	No	No	No	No	During Urin...
212	No	No	No	No	No	During Urin...
213	No	No	No	No	No	During Urin...
214	No	No	No	No	No	During Urin...
215	No	No	No	Pill	No	During Urin...
216	No	No	No	Implant	No	During Urin...
217	No	No	No	No	Yes	During Urin...
218	No	No	No	No	No	During Urin...
219	No	No	No	No	Yes	During Urin...
220	No	No	No	Pill	No	During Urin...
221	No	No	No	No	No	During Urin...
222	No	No	No	Implant	No	during devi...
223	No	No	No	No	No	during devi...
224	No	No	No	No	No	every morni...
225	No	No	No	No	No	During Urin...
226	No	No	No	No	No	during devi...
227	No	No	No	Pill	No	During Urin...
228	No	No	No	No	No	others
229	No	No	No	No	No	During Urin...
230	No	No	No	Condom	Yes	during devi...
231	No	No	No	No	No	during devi...
232	No	No	No	Condom	No	during devi...
233	No	No	No	No	No	during devi...
234	No	No	No	Implant	No	During Urin...
235	No	No	No	Pill	No	every morni...
236	No	No	No	Pill	Yes	During Urin...
237	No	No	No	No	No	During Urin...
238	No	No	No	No	No	During Urin...
239	No	No	No	No	No	During Urin...
240	No	No	No	IUD	Yes	During Urin...
241	No	No	No	No	No	others
242	No	No	No	Injection	No	during devi...
243	No	No	No	Implant	No	During Urin...
244	No	No	No	No	No	during devi...
245	No	No	No	No	No	during devi...

	SexFrequency	SU	BactSpp	FungSpp	CL	E
211	NO	Postive	S.aureus	Candidia al...	Sensetive	.
212	NO	Negative	.	.	.	.
213	<3 times	Postive	.	Candidia tr...	.	.
214	NO	Negative	.	.	.	.
215	<3 times	Negative	.	.	.	.
216	NO	Negative	.	.	.	.
217	NO	Negative	.	.	.	.
218	NO	Negative	.	.	.	.
219	NO	Postive	S. saproph...	Candidia tr...	Sensetive	.
220	NO	Negative	.	.	.	.
221	NO	Postive	.	Candidia al...	.	.
222	<3 times	Negative	.	.	.	.
223	NO	Negative	.	.	.	.
224	NO	Postive	S.aureus	.	Sensetive	Sensetive
225	NO	Negative	.	.	.	.
226	NO	Postive	.	Candidia kr...	.	.
227	<3 times	Negative	.	.	.	.
228	NO	Negative	.	.	.	.
229	NO	Negative	.	.	.	.
230	NO	Negative	.	.	.	.
231	NO	Negative	.	.	.	.
232	NO	Postive	Citrobacter	.	.	.
233	NO	Negative	.	.	.	.
234	NO	Negative	.	.	.	.
235	NO	Postive	.	Candidia al...	.	.
236	<3 times	Postive	Klebsella r...	.	.	.
237	NO	Negative	.	.	.	.
238	NO	Negative	.	.	.	.
239	NO	Negative	.	.	.	.
240	NO	Negative	.	.	.	.
241	NO	Negative	.	.	.	.
242	<3 times	Negative	.	.	.	.
243	<3 times	Negative	.	.	.	.
244	NO	Negative	.	.	.	.
245	>=3 times	Negative	.	.	.	.

	PEN	CAF	CIP	TTC	SXT	F
211	Resistant	Sensetive	Sensetive	Resistant	Resistant	Sensetive
212	.	.	.	.	.	.
213	.	.	.	.	.	.
214	.	.	.	.	.	.
215	.	.	.	.	.	.
216	.	.	.	.	.	.
217	.	.	.	.	.	.
218	.	.	.	.	.	.
219	Resistant	Resistant	Sensetive	Sensetive	Resistant	Sensetive
220	.	.	.	.	.	.
221	.	.	.	.	.	.
222	.	.	.	.	.	.
223	.	.	.	.	.	.
224	Resistant	Sensetive	Sensetive	Sensetive	Resistant	Sensetive
225	.	.	.	.	.	.
226	.	.	.	.	.	.
227	.	.	.	.	.	.
228	.	.	.	.	.	.
229	.	.	.	.	.	.
230	.	.	.	.	.	.
231	.	.	.	.	.	.
232	.	.	Intermidate	Sensetive	Sensetive	Sensetive
233	.	.	.	.	.	.
234	.	.	.	.	.	.
235	.	.	.	.	.	.
236	.	.	Sensetive	Resistant	Resistant	Sensetive
237	.	.	.	.	.	.
238	.	.	.	.	.	.
239	.	.	.	.	.	.
240	.	.	.	.	.	.
241	.	.	.	.	.	.
242	.	.	.	.	.	.
243	.	.	.	.	.	.
244	.	.	.	.	.	.
245	.	.	.	.	.	.

	NOR	CRO	AMC	CTX	CAZ	AMP
211	Sensetive	.	.	.	.	.
212	.	.	.	.	.	.
213	.	.	.	.	.	.
214	.	.	.	.	.	.
215	.	.	.	.	.	.
216	.	.	.	.	.	.
217	.	.	.	.	.	.
218	.	.	.	.	.	.
219	Sensetive	.	.	.	.	.
220	.	.	.	.	.	.
221	.	.	.	.	.	.
222	.	.	.	.	.	.
223	.	.	.	.	.	.
224	Sensetive	.	.	.	.	.
225	.	.	.	.	.	.
226	.	.	.	.	.	.
227	.	.	.	.	.	.
228	.	.	.	.	.	.
229	.	.	.	.	.	.
230	.	.	.	.	.	.
231	.	.	.	.	.	.
232	Sensetive	Sensetive	Resistant	Sensetive	Resistant	Intermidate
233	.	.	.	.	.	.
234	.	.	.	.	.	.
235	.	.	.	.	.	.
236	Sensetive	Resistant	Resistant	Intermidate	Sensetive	.
237	.	.	.	.	.	.
238	.	.	.	.	.	.
239	.	.	.	.	.	.
240	.	.	.	.	.	.
241	.	.	.	.	.	.
242	.	.	.	.	.	.
243	.	.	.	.	.	.
244	.	.	.	.	.	.
245	.	.	.	.	.	.

	AMK	GEN	ResPattern	MDR	GENTTWASH
211	.	.	R3	Yes	After urination
212	.	.	.	.	After urination
213	.	.	.	.	After urination
214	.	.	.	.	After defication
215	.	.	.	.	After urination
216	.	.	.	.	After urination
217	.	.	.	.	After defication
218	.	.	.	.	After urination
219	.	.	R3	Yes	After urination
220	.	.	.	.	After urination
221	.	.	.	.	After urination
222	.	.	.	.	After defication
223	.	.	.	.	After defication
224	.	.	R2	No	After urination
225	.	.	.	.	After urination
226	.	.	.	.	After defication
227	.	.	.	.	After urination
228	.	.	.	.	After defication
229	.	.	.	.	After urination
230	.	.	.	.	After defication
231	.	.	.	.	After defication
232	Sensetive	Sensetive	R4	Yes	After defication
233	.	.	.	.	After defication
234	.	.	.	.	After urination
235	.	.	.	.	After urination
236	Sensetive	Sensetive	>=R5	Yes	After urination
237	.	.	.	.	After urination
238	.	.	.	.	After urination
239	.	.	.	.	After urination
240	.	.	.	.	After urination
241	.	.	.	.	After defication
242	.	.	.	.	After defication
243	.	.	.	.	After urination
244	.	.	.	.	After defication
245	.	.	.	.	After defication

spss data Bre.sav

	ID	Age	AgeDscrptve	college	Residence	Department
246	32	28	26-30	Alkan Healt...	Urban	Health Scie...
247	76	18	15-20	Alkan Healt...	Urban	Health Scie...
248	81	20	15-20	Alkan Healt...	Urban	Health Scie...
249	11	22	21-25	Alkan Healt...	Urban	Health Scie...
250	6	19	15-20	Alkan Healt...	Urban	Health Scie...
251	43	22	21-25	Alkan Healt...	Urban	Health Scie...
252	56	20	15-20	Alkan Healt...	Urban	Health Scie...
253	71	20	15-20	Alkan Healt...	Urban	Health Scie...
254	77	18	15-20	Alkan Healt...	Urban	Health Scie...
255	26	20	15-20	Alkan Healt...	Urban	Health Scie...
256	48	19	15-20	Alkan Healt...	Urban	Health Scie...
257	37	19	15-20	Alkan Healt...	Urban	Health Scie...
258	21	20	15-20	Alkan Healt...	Urban	Health Scie...
259	30	21	21-25	Alkan Healt...	Urban	Health Scie...
260	106	19	15-20	Alkan Healt...	Urban	Social Scie...
261	91	21	21-25	Alkan Healt...	Urban	Social Scie...
262	83	19	15-20	Alkan Healt...	Urban	Social Scie...
263	71	20	15-20	Alkan Healt...	Urban	Social Scie...
264	69	25	21-25	Alkan Healt...	Urban	Social Scie...
265	55	25	21-25	Alkan Healt...	Urban	Social Scie...
266	43	19	15-20	Alkan Healt...	Urban	Social Scie...
267	36	18	15-20	Alkan Healt...	Urban	Social Scie...
268	77	22	21-25	Alkan Healt...	Urban	Social Scie...
269	32	20	15-20	Alkan Healt...	Urban	Social Scie...
270	21	23	21-25	Dessie Hea...	Urban	Health Scie...
271	16	20	15-20	Dessie Hea...	Urban	Health Scie...
272	25	20	15-20	Dessie Hea...	Urban	Health Scie...
273	92	21	21-25	Dessie Hea...	Urban	Health Scie...
274	86	23	21-25	Dessie Hea...	Urban	Health Scie...
275	28	20	15-20	Dessie Hea...	Urban	Health Scie...
276	73	24	21-25	Dessie Hea...	Urban	Health Scie...
277	64	22	21-25	Dessie Hea...	Urban	Health Scie...
278	91	22	21-25	Dessie Hea...	Urban	Health Scie...
279	29	20	15-20	Dessie Hea...	Urban	Health Scie...
280	78	20	15-20	Dessie Hea...	Urban	Health Scie...

	Batch	MartStatus	Income	Catheter	DM	HIV
246	Third Year	Married	>4000	No	No	No
247	Third Year	Single	3001-4000	No	No	No
248	Third Year	Single	>4000	No	No	No
249	Third Year	Married	>4000	No	No	No
250	Third Year	Single	>4000	No	No	No
251	Third Year	Divorced	3001-4000	No	No	No
252	Third Year	Single	≤1000	No	No	No
253	Third Year	Single	>4000	No	No	No
254	Third Year	Married	1001-2000	No	No	No
255	Third Year	Single	>4000	No	No	No
256	Third Year	Single	≤1000	No	No	No
257	Third Year	Single	>4000	No	No	No
258	Third Year	Single	>4000	No	No	No
259	Third Year	Single	>4000	No	No	No
260	Third Year	Single	>4000	No	No	No
261	Third Year	Single	>4000	No	No	No
262	Third Year	Single	2001-3000	No	No	No
263	Third Year	Single	>4000	No	No	No
264	Third Year	Single	2001-3000	No	No	No
265	Third Year	Married	>4000	No	No	No
266	Third Year	Single	>4000	No	No	No
267	Third Year	Single	>4000	No	No	No
268	Third Year	Single	2001-3000	No	No	No
269	Third Year	Single	>4000	No	No	No
270	Third Year	Married	>4000	No	No	No
271	Third Year	Married	3001-4000	No	No	No
272	Third Year	Single	>4000	No	No	Yes
273	Third Year	Single	3001-4000	No	No	No
274	Third Year	Single	>4000	No	No	No
275	Third Year	Single	>4000	No	No	No
276	Third Year	Single	1001-2000	No	No	No
277	Third Year	Single	≤1000	No	No	No
278	Third Year	Single	1001-2000	No	No	No
279	Third Year	Single	>4000	No	No	No
280	Third Year	Single	>4000	No	No	Yes

	STD	Hospitalization	Antibiotics	Contracepive	GentAbnorm	GentWash
246	No	No	No	Pill	Yes	every morni...
247	No	No	No	Injection	No	during devi...
248	No	No	No	Implant	No	every morni...
249	No	No	No	Pill	No	During Urin...
250	No	No	No	No	No	During Urin...
251	No	No	No	Pill	No	During Urin...
252	No	No	No	Pill	No	During Urin...
253	No	No	No	Injection	No	During Urin...
254	No	No	No	Injection	No	during devi...
255	No	No	No	No	No	During Urin...
256	No	No	No	No	Yes	during devi...
257	No	No	No	No	Yes	During Urin...
258	No	No	No	No	No	During Urin...
259	No	No	No	Condom	No	During Urin...
260	No	No	No	No	No	every morni...
261	No	No	No	No	No	During Urin...
262	No	No	No	No	No	During Urin...
263	No	No	No	No	No	During Urin...
264	No	No	No	No	No	During Urin...
265	No	No	No	Injection	No	During Urin...
266	No	No	No	No	No	During Urin...
267	No	No	No	No	No	During Urin...
268	No	No	No	Condom	No	every morni...
269	No	No	No	No	No	During Urin...
270	No	No	No	IUD	No	others
271	No	No	No	No	No	every morni...
272	No	No	No	No	No	every morni...
273	No	No	No	No	No	During Urin...
274	No	No	No	Injection	No	During Urin...
275	No	No	No	No	No	During Urin...
276	No	No	No	No	No	During Urin...
277	No	No	No	No	No	During Urin...
278	No	No	No	No	No	During Urin...
279	No	No	No	Injection	No	During Urin...
280	No	No	No	No	No	During Urin...

	SexFrequency	SU	BactSpp	FungSpp	CL	E
246	<3 times	Postive	.	Candidia al...	.	.
247	<3 times	Negative	.	.	.	.
248	<3 times	Postive	S. saproph...	.	Sensetive	Intermidate
249	>=3 times	Negative	.	.	.	.
250	NO	Negative	.	.	.	.
251	NO	Negative	.	.	.	.
252	<3 times	Negative	.	.	.	.
253	<3 times	Negative	.	.	.	.
254	<3 times	Negative	.	.	.	.
255	>=3 times	Negative	.	.	.	.
256	NO	Negative	.	.	.	.
257	NO	Negative	.	.	.	.
258	>=3 times	Negative	.	.	.	.
259	NO	Negative	.	.	.	.
260	NO	Negative	.	.	.	.
261	>=3 times	Negative	.	.	.	.
262	NO	Negative	.	.	.	.
263	NO	Negative	.	.	.	.
264	NO	Negative	.	.	.	.
265	<3 times	Negative	.	.	.	.
266	NO	Negative	.	.	.	.
267	NO	Negative	.	.	.	.
268	NO	Negative	.	.	.	.
269	NO	Negative	.	.	.	.
270	<3 times	Negative	.	.	.	.
271	<3 times	Negative	.	.	.	.
272	>=3 times	Postive	Klebsella o...	Candidia gl...	.	.
273	NO	Negative	.	.	.	.
274	<3 times	Negative	.	.	.	.
275	NO	Negative	.	.	.	.
276	NO	Negative	.	.	.	.
277	>=3 times	Negative	.	.	.	.
278	NO	Negative	.	.	.	.
279	NO	Negative	.	.	.	.
280	NO	Negative	.	.	.	.

	PEN	CAF	CIP	TTC	SXT	F
246	.	.	.	.	.	.
247	.	.	.	.	.	.
248	Resistant	Resistant	Intermidate	Resistant	Resistant	Sensetive
249	.	.	.	.	.	.
250	.	.	.	.	.	.
251	.	.	.	.	.	.
252	.	.	.	.	.	.
253	.	.	.	.	.	.
254	.	.	.	.	.	.
255	.	.	.	.	.	.
256	.	.	.	.	.	.
257	.	.	.	.	.	.
258	.	.	.	.	.	.
259	.	.	.	.	.	.
260	.	.	.	.	.	.
261	.	.	.	.	.	.
262	.	.	.	.	.	.
263	.	.	.	.	.	.
264	.	.	.	.	.	.
265	.	.	.	.	.	.
266	.	.	.	.	.	.
267	.	.	.	.	.	.
268	.	.	.	.	.	.
269	.	.	.	.	.	.
270	.	.	.	.	.	.
271	.	.	.	.	.	.
272	.	.	Sensetive	Intermidate	Resistant	Resistant
273	.	.	.	.	.	.
274	.	.	.	.	.	.
275	.	.	.	.	.	.
276	.	.	.	.	.	.
277	.	.	.	.	.	.
278	.	.	.	.	.	.
279	.	.	.	.	.	.
280	.	.	.	.	.	.

	NOR	CRO	AMC	CTX	CAZ	AMP
246	.	.	.	.	.	.
247	.	.	.	.	.	.
248	Sensetive	.	.	.	.	.
249	.	.	.	.	.	.
250	.	.	.	.	.	.
251	.	.	.	.	.	.
252	.	.	.	.	.	.
253	.	.	.	.	.	.
254	.	.	.	.	.	.
255	.	.	.	.	.	.
256	.	.	.	.	.	.
257	.	.	.	.	.	.
258	.	.	.	.	.	.
259	.	.	.	.	.	.
260	.	.	.	.	.	.
261	.	.	.	.	.	.
262	.	.	.	.	.	.
263	.	.	.	.	.	.
264	.	.	.	.	.	.
265	.	.	.	.	.	.
266	.	.	.	.	.	.
267	.	.	.	.	.	.
268	.	.	.	.	.	.
269	.	.	.	.	.	.
270	.	.	.	.	.	.
271	.	.	.	.	.	.
272	Sensetive	Resistant	Resistant	Resistant	Resistant	.
273	.	.	.	.	.	.
274	.	.	.	.	.	.
275	.	.	.	.	.	.
276	.	.	.	.	.	.
277	.	.	.	.	.	.
278	.	.	.	.	.	.
279	.	.	.	.	.	.
280	.	.	.	.	.	.

	AMK	GEN	ResPattern	MDR	GENTTWASH
246	.	.	.	.	Afterurination
247	.	.	.	.	Afterdefication
248	.	.	>=R5	Yes	Afterurination
249	.	.	.	.	Afterurination
250	.	.	.	.	Afterurination
251	.	.	.	.	Afterurination
252	.	.	.	.	Afterurination
253	.	.	.	.	Afterurination
254	.	.	.	.	Afterdefication
255	.	.	.	.	Afterurination
256	.	.	.	.	Afterdefication
257	.	.	.	.	Afterurination
258	.	.	.	.	Afterurination
259	.	.	.	.	Afterurination
260	.	.	.	.	Afterurination
261	.	.	.	.	Afterurination
262	.	.	.	.	Afterurination
263	.	.	.	.	Afterurination
264	.	.	.	.	Afterurination
265	.	.	.	.	Afterurination
266	.	.	.	.	Afterurination
267	.	.	.	.	Afterurination
268	.	.	.	.	Everymorning
269	.	.	.	.	Afterurination
270	.	.	.	.	Afterdefication
271	.	.	.	.	Everymorning
272	Sensetive	Sensetive	>=R5	Yes	Afterurination
273	.	.	.	.	Afterurination
274	.	.	.	.	Afterurination
275	.	.	.	.	Afterurination
276	.	.	.	.	Afterdefication
277	.	.	.	.	Afterurination
278	.	.	.	.	Afterurination
279	.	.	.	.	Afterurination
280	.	.	.	.	Afterurination

	ID	Age	AgeDscrptve	college	Residence	Department
281	103	20	15-20	Dessie Hea...	Urban	Health Scie...
282	15	20	15-20	Dessie Hea...	Urban	Health Scie...
283	94	20	15-20	Dessie Hea...	Urban	Health Scie...
284	29	20	15-20	Dessie Hea...	Rural	Health Scie...
285	18	20	15-20	Dessie Hea...	Urban	Health Scie...
286	21	23	21-25	Dessie Hea...	Urban	Health Scie...
287	153	19	15-20	Dessie Hea...	Urban	Health Scie...
288	60	22	21-25	Dessie Hea...	Urban	Health Scie...
289	9	20	15-20	Dessie Hea...	Urban	Health Scie...
290	12	19	15-20	Dessie Hea...	Urban	Health Scie...
291	16	20	15-20	Dessie Hea...	Urban	Health Scie...
292	18	20	15-20	Dessie Hea...	Urban	Health Scie...
293	29	21	21-25	Dessie Hea...	Urban	Health Scie...
294	23	22	21-25	Dessie Hea...	Urban	Health Scie...
295	16	21	21-25	Dessie Hea...	Urban	Health Scie...
296	19	19	15-20	Dessie Hea...	Urban	Health Scie...
297	31	23	21-25	Dessie Hea...	Urban	Health Scie...
298	33	18	15-20	Dessie Hea...	Urban	Health Scie...
299	36	22	21-25	Dessie Hea...	Urban	Health Scie...
300	38	24	21-25	Dessie Hea...	Urban	Health Scie...
301	39	22	21-25	Dessie Hea...	Urban	Health Scie...
302	42	23	21-25	Dessie Hea...	Urban	Health Scie...
303	46	19	15-20	Dessie Hea...	Urban	Health Scie...
304	49	23	21-25	Dessie Hea...	Urban	Health Scie...
305	51	20	15-20	Dessie Hea...	Urban	Health Scie...
306	53	29	26-30	Dessie Hea...	Urban	Health Scie...
307	57	19	15-20	Dessie Hea...	Urban	Health Scie...
308	59	20	15-20	Dessie Hea...	Rural	Health Scie...
309	66	19	15-20	Dessie Hea...	Urban	Health Scie...
310	63	20	15-20	Dessie Hea...	Urban	Health Scie...
311	68	19	15-20	Dessie Hea...	Rural	Health Scie...
312	86	21	21-25	Dessie Hea...	Urban	Health Scie...
313	83	19	15-20	Dessie Hea...	Urban	Health Scie...
314	88	20	15-20	Dessie Hea...	Rural	Health Scie...
315	76	19	15-20	Dessie Hea...	Urban	Health Scie...

	Batch	MartStatus	Income	Catheter	DM	HIV
281	Third Year	Single	>4000	No	No	No
282	Third Year	Single	≤1000	No	No	No
283	Third Year	Single	1001-2000	No	No	No
284	Third Year	Single	≤1000	No	No	No
285	Third Year	Single	3001-4000	No	No	No
286	Third Year	Married	3001-4000	No	No	No
287	Third Year	Single	>4000	No	No	Yes
288	Third Year	Divorced	2001-3000	No	No	No
289	Third Year	Married	>4000	No	No	No
290	Third Year	Married	>4000	No	No	No
291	Third Year	Single	>4000	No	No	No
292	Third Year	Single	1001-2000	No	No	No
293	Third Year	Single	1001-2000	No	No	No
294	Third Year	Single	1001-2000	No	No	No
295	Third Year	Divorced	≤1000	No	No	No
296	Third Year	Single	2001-3000	No	No	No
297	Third Year	Single	≤1000	No	No	No
298	Third Year	Single	1001-2000	No	No	No
299	Third Year	Single	1001-2000	No	No	Yes
300	Third Year	Single	1001-2000	No	No	No
301	Third Year	Single	>4000	No	No	No
302	Third Year	Divorced	>4000	No	No	No
303	Third Year	Single	>4000	No	No	No
304	Third Year	Married	1001-2000	No	No	No
305	Third Year	Married	1001-2000	No	No	No
306	Third Year	Married	>4000	No	No	No
307	Third Year	Married	>4000	No	No	No
308	Third Year	Single	>4000	No	No	No
309	Third Year	Single	3001-4000	No	No	No
310	Third Year	Single	>4000	No	No	No
311	Third Year	Single	3001-4000	No	No	No
312	Third Year	Single	>4000	No	No	No
313	Third Year	Single	3001-4000	No	No	No
314	Third Year	Single	3001-4000	No	No	No
315	Third Year	Single	1001-2000	No	No	No

	STD	Hospitalization	Antibiotics	Contracepive	GentAbnorm	GentWash
281	No	No	No	No	No	every morni...
282	No	No	No	No	No	During Urin...
283	No	No	No	Injection	No	During Urin...
284	No	No	No	Injection	No	During Urin...
285	No	No	No	No	No	during devi...
286	No	No	No	Implant	No	During Urin...
287	No	No	No	Condom	No	others
288	No	No	No	Implant	No	During Urin...
289	No	No	No	Injection	No	others
290	No	No	No	Pill	No	during devi...
291	No	No	No	Injection	No	during devi...
292	No	No	No	Implant	No	during devi...
293	No	No	No	No	No	During Urin...
294	No	No	No	No	No	every morni...
295	No	No	No	No	No	every morni...
296	No	No	No	No	No	During Urin...
297	No	No	No	Injection	No	every morni...
298	No	No	No	No	No	every morni...
299	No	No	No	No	No	During Urin...
300	No	No	No	No	No	during devi...
301	No	No	No	No	No	others
302	No	No	No	Implant	No	during devi...
303	No	No	No	No	No	During Urin...
304	No	No	No	Injection	No	During Urin...
305	No	No	No	Implant	No	During Urin...
306	No	No	No	Condom	No	During Urin...
307	No	No	No	Implant	Yes	During Urin...
308	No	No	No	No	No	During Urin...
309	No	No	No	Condom	No	During Urin...
310	No	No	No	No	No	every morni...
311	No	No	No	No	No	During Urin...
312	No	Yes	Yes	Implant	No	During Urin...
313	No	No	No	No	No	During Urin...
314	No	No	No	No	No	during devi...
315	No	No	No	Implant	No	during devi...

	SexFrequency	SU	BactSpp	FungSpp	CL	E
281	NO	Negative	.	.	.	.
282	NO	Negative	.	.	.	.
283	NO	Negative	.	.	.	.
284	<3 times	Negative	.	.	.	.
285	NO	Negative	.	.	.	.
286	NO	Negative	.	.	.	.
287	>=3 times	Postive	E.coli	.	.	.
288	NO	Negative	.	.	.	.
289	>=3 times	Negative	.	.	.	.
290	NO	Postive	.	Candidia tr...	.	.
291	NO	Negative	.	.	.	.
292	<3 times	Negative	.	.	.	.
293	NO	Negative	.	.	.	.
294	NO	Negative	.	.	.	.
295	<3 times	Postive	S. saproph...	.	Sensetive	Intermidate
296	NO	Negative	.	.	.	.
297	<3 times	Negative	.	.	.	.
298	NO	Negative	.	.	.	.
299	NO	Negative	.	.	.	.
300	NO	Negative	.	.	.	.
301	NO	Negative	.	.	.	.
302	NO	Postive	.	Candidia gl...	.	.
303	NO	Negative	.	.	.	.
304	NO	Negative	.	.	.	.
305	<3 times	Negative	.	.	.	.
306	>=3 times	Postive	.	Candidia al...	.	.
307	<3 times	Negative	.	.	.	.
308	NO	Negative	.	.	.	.
309	NO	Negative	.	.	.	.
310	NO	Negative	.	.	.	.
311	NO	Negative	.	.	.	.
312	<3 times	Negative	.	.	.	.
313	NO	Negative	.	.	.	.
314	NO	Postive	Klebsella o...	.	.	.
315	NO	Negative	.	.	.	.

	PEN	CAF	CIP	TTC	SXT	F
281	.	.	.	.	.	.
282	.	.	.	.	.	.
283	.	.	.	.	.	.
284	.	.	.	.	.	.
285	.	.	.	.	.	.
286	.	.	.	.	.	.
287	.	.	Sensetive	Resistant	Sensetive	Sensetive
288	.	.	.	.	.	.
289	.	.	.	.	.	.
290	.	.	.	.	.	.
291	.	.	.	.	.	.
292	.	.	.	.	.	.
293	.	.	.	.	.	.
294	.	.	.	.	.	.
295	Resistant	Resistant	Sensetive	Resistant	Resistant	Sensetive
296	.	.	.	.	.	.
297	.	.	.	.	.	.
298	.	.	.	.	.	.
299	.	.	.	.	.	.
300	.	.	.	.	.	.
301	.	.	.	.	.	.
302	.	.	.	.	.	.
303	.	.	.	.	.	.
304	.	.	.	.	.	.
305	.	.	.	.	.	.
306	.	.	.	.	.	.
307	.	.	.	.	.	.
308	.	.	.	.	.	.
309	.	.	.	.	.	.
310	.	.	.	.	.	.
311	.	.	.	.	.	.
312	.	.	.	.	.	.
313	.	.	.	.	.	.
314	.	.	Sensetive	Intermidate	Resistant	Resistant
315	.	.	.	.	.	.

	NOR	CRO	AMC	CTX	CAZ	AMP
281	.	.	.	.	.	.
282	.	.	.	.	.	.
283	.	.	.	.	.	.
284	.	.	.	.	.	.
285	.	.	.	.	.	.
286	.	.	.	.	.	.
287	Sensetive	Sensetive	Resistant	Sensetive	Sensetive	Resistant
288	.	.	.	.	.	.
289	.	.	.	.	.	.
290	.	.	.	.	.	.
291	.	.	.	.	.	.
292	.	.	.	.	.	.
293	.	.	.	.	.	.
294	.	.	.	.	.	.
295	Sensetive	.	.	.	.	.
296	.	.	.	.	.	.
297	.	.	.	.	.	.
298	.	.	.	.	.	.
299	.	.	.	.	.	.
300	.	.	.	.	.	.
301	.	.	.	.	.	.
302	.	.	.	.	.	.
303	.	.	.	.	.	.
304	.	.	.	.	.	.
305	.	.	.	.	.	.
306	.	.	.	.	.	.
307	.	.	.	.	.	.
308	.	.	.	.	.	.
309	.	.	.	.	.	.
310	.	.	.	.	.	.
311	.	.	.	.	.	.
312	.	.	.	.	.	.
313	.	.	.	.	.	.
314	Sensetive	Resistant	Resistant	Resistant	Resistant	.
315	.	.	.	.	.	.

spss data Bre.sav

	AMK	GEN	ResPattern	MDR	GENTTWASH
281	.	.	.	.	Afterurination
282	.	.	.	.	Afterurination
283	.	.	.	.	Afterdefication
284	.	.	.	.	Afterdefication
285	.	.	.	.	Afterdefication
286	.	.	.	.	Afterurination
287	Sensetive	Sensetive	R3	Yes	Others
288	.	.	.	.	Afterurination
289	.	.	.	.	Others
290	.	.	.	.	Afterdefication
291	.	.	.	.	Afterdefication
292	.	.	.	.	Afterdefication
293	.	.	.	.	Afterurination
294	.	.	.	.	Everymorning
295	.	.	R4	Yes	Everymorning
296	.	.	.	.	Afterurination
297	.	.	.	.	Everymorning
298	.	.	.	.	Afterurination
299	.	.	.	.	Afterurination
300	.	.	.	.	Afterdefication
301	.	.	.	.	Afterdefication
302	.	.	.	.	Afterurination
303	.	.	.	.	Afterurination
304	.	.	.	.	Afterurination
305	.	.	.	.	Afterurination
306	.	.	.	.	Afterurination
307	.	.	.	.	Afterurination
308	.	.	.	.	Afterurination
309	.	.	.	.	Afterurination
310	.	.	.	.	Afterurination
311	.	.	.	.	Afterurination
312	.	.	.	.	Afterurination
313	.	.	.	.	Afterurination
314	Sensetive	Sensetive	>=R5	Yes	Afterdefication
315	.	.	.	.	Afterdefication

	ID	Age	AgeDscrptve	college	Residence	Department
316	79	21	21-25	Dessie Hea...	Urban	Health Scie...
317	73	20	15-20	Dessie Hea...	Rural	Health Scie...
318	71	23	21-25	Dessie Hea...	Rural	Health Scie...
319	103	22	21-25	Dessie Hea...	Urban	Health Scie...
320	109	20	15-20	Dessie Hea...	Rural	Health Scie...
321	186	20	15-20	Dessie Hea...	Urban	Health Scie...
322	93	37	>30	Dessie Hea...	Urban	Health Scie...
323	97	23	21-25	Dessie Hea...	Urban	Health Scie...
324	91	20	15-20	Dessie Hea...	Rural	Health Scie...
325	141	21	21-25	Dessie Hea...	Rural	Health Scie...
326	143	20	15-20	Dessie Hea...	Urban	Health Scie...
327	12	23	21-25	Alkan Healt...	Urban	Health Scie...
328	19	24	21-25	Alkan Healt...	Urban	Health Scie...
329	31	22	21-25	Alkan Healt...	Urban	Health Scie...
330	28	27	26-30	Alkan Healt...	Urban	Health Scie...
331	39	23	21-25	Alkan Healt...	Urban	Health Scie...
332	46	22	21-25	Alkan Healt...	Urban	Health Scie...
333	40	23	21-25	Alkan Healt...	Urban	Health Scie...
334	29	24	21-25	Alkan Healt...	Urban	Health Scie...
335	33	22	21-25	Alkan Healt...	Urban	Health Scie...
336	56	31	>30	Alkan Healt...	Urban	Health Scie...
337	62	23	21-25	Alkan Healt...	Urban	Health Scie...
338	77	22	21-25	Alkan Healt...	Urban	Health Scie...
339	89	30	26-30	Alkan Healt...	Urban	Health Scie...
340	91	24	21-25	Alkan Healt...	Urban	Health Scie...
341	12	28	26-30	Alkan Healt...	Urban	Health Scie...
342	8	25	21-25	Alkan Healt...	Urban	Health Scie...
343	98	26	26-30	Alkan Healt...	Urban	Health Scie...
344	32	30	26-30	Alkan Healt...	Urban	Health Scie...
345	94	23	21-25	Alkan Healt...	Urban	Health Scie...
346	80	33	>30	Alkan Healt...	Urban	Health Scie...
347	43	25	21-25	Alkan Healt...	Urban	Health Scie...
348	32	31	>30	Alkan Healt...	Urban	Health Scie...
349	21	24	21-25	Alkan Healt...	Urban	Health Scie...
350	15	21	21-25	Dessie coll...	Urban	Language

	Batch	MartStatus	Income	Catheter	DM	HIV
316	Third Year	Divorced	≤1000	No	No	No
317	Third Year	Single	1001-2000	No	No	No
318	Third Year	Single	≤1000	No	No	No
319	Third Year	Single	1001-2000	No	No	No
320	Third Year	Single	2001-3000	No	No	No
321	Third Year	Single	>4000	No	No	No
322	Third Year	Single	>4000	No	No	No
323	Third Year	Married	3001-4000	No	No	No
324	Third Year	Single	2001-3000	No	No	No
325	Third Year	Single	>4000	No	No	No
326	Third Year	Single	1001-2000	No	No	No
327	Fourth Year	Married	>4000	No	Yes	No
328	Fourth Year	Single	1001-2000	No	No	No
329	Fourth Year	Single	>4000	No	No	No
330	Fourth Year	Single	>4000	No	No	No
331	Fourth Year	Single	>4000	No	No	No
332	Fourth Year	Single	1001-2000	No	No	No
333	Fourth Year	Single	3001-4000	Yes	Yes	No
334	Fourth Year	Single	>4000	No	No	No
335	Fourth Year	Single	>4000	No	No	No
336	Fourth Year	Married	>4000	No	No	No
337	Fourth Year	Single	>4000	No	No	No
338	Fourth Year	Single	>4000	Yes	No	No
339	Fourth Year	Married	>4000	No	No	No
340	Fourth Year	Single	>4000	No	No	No
341	Fourth Year	Married	>4000	No	No	No
342	Fourth Year	Married	>4000	Yes	No	No
343	Fourth Year	Single	3001-4000	No	No	No
344	Fourth Year	Single	>4000	No	No	No
345	Fourth Year	Single	>4000	No	No	No
346	Fourth Year	Married	>4000	No	No	No
347	Fourth Year	Married	>4000	No	No	No
348	Fourth Year	Married	>4000	No	No	No
349	Fourth Year	Single	>4000	No	No	No
350	Second Year	Single	3001-4000	No	No	No

	STD	Hospitalization	Antibiotics	Contracepive	GentAbnorm	GentWash
316	No	No	No	Condom	No	During Urin...
317	No	Yes	Yes	No	No	during devi...
318	No	No	No	No	No	others
319	No	No	No	No	No	during devi...
320	No	No	No	No	No	During Urin...
321	No	No	No	No	No	others
322	No	No	No	IUD	No	During Urin...
323	No	No	No	Implant	No	During Urin...
324	No	No	No	Condom	No	During Urin...
325	No	No	No	Implant	No	during devi...
326	No	No	No	No	No	during devi...
327	No	No	No	Pill	No	During Urin...
328	No	No	No	No	No	During Urin...
329	Yes	No	No	No	Yes	others
330	No	No	No	Pill	No	During Urin...
331	No	No	Yes	No	No	others
332	No	Yes	Yes	Pill	No	every morni...
333	No	No	No	Pill	No	during devi...
334	No	Yes	Yes	No	No	During Urin...
335	No	No	No	No	No	During Urin...
336	No	No	No	Pill	No	During Urin...
337	No	No	No	No	No	During Urin...
338	No	Yes	Yes	No	No	During Urin...
339	No	No	No	Pill	No	others
340	No	No	No	No	No	During Urin...
341	No	No	No	No	Yes	During Urin...
342	No	No	No	Implant	No	during devi...
343	No	No	No	No	No	others
344	No	No	Yes	Pill	No	During Urin...
345	No	Yes	Yes	No	No	During Urin...
346	No	No	Yes	Condom	No	During Urin...
347	No	No	No	No	No	During Urin...
348	No	No	No	No	No	During Urin...
349	No	No	No	IUD	Yes	others
350	No	Yes	Yes	No	No	During Urin...

spss data Bre.sav

	SexFrequency	SU	BactSpp	FungSpp	CL	E
316	NO	Negative	.	.	.	.
317	>=3 times	Postive	S.aureus	Candidia tr...	Intermideate	.
318	NO	Negative	.	.	.	.
319	NO	Negative	.	.	.	.
320	NO	Negative	.	.	.	.
321	NO	Negative	.	.	.	.
322	NO	Negative	.	.	.	.
323	<3 times	Negative	.	.	.	.
324	NO	Negative	.	.	.	.
325	NO	Postive	.	Candidia tr...	.	.
326	NO	Postive	S. saproph...	.	Sensetive	Intermidate
327	NO	Negative	.	.	.	.
328	NO	Negative	.	.	.	.
329	NO	Postive	.	Candidia kr...	.	.
330	NO	Negative	.	.	.	.
331	NO	Negative	.	.	.	.
332	NO	Negative	.	.	.	.
333	NO	Negative	.	.	.	.
334	NO	Negative	.	.	.	.
335	NO	Negative	.	.	.	.
336	<3 times	Negative	.	.	.	.
337	NO	Negative	.	.	.	.
338	NO	Negative	.	.	.	.
339	<3 times	Negative	.	.	.	.
340	NO	Negative	.	.	.	.
341	<3 times	Negative	.	.	.	.
342	<3 times	Negative	.	.	.	.
343	NO	Negative	.	.	.	.
344	NO	Negative	.	.	.	.
345	NO	Negative	.	.	.	.
346	>=3 times	Negative	.	.	.	.
347	NO	Negative	.	.	.	.
348	NO	Negative	.	.	.	.
349	NO	Negative	.	.	.	.
350	NO	Negative	.	.	.	.

	PEN	CAF	CIP	TTC	SXT	F
316	.	.	.	.	.	.
317	Resistant	Sensetive	Intermidate	Sensetive	Resistant	Sensetive
318	.	.	.	.	.	.
319	.	.	.	.	.	.
320	.	.	.	.	.	.
321	.	.	.	.	.	.
322	.	.	.	.	.	.
323	.	.	.	.	.	.
324	.	.	.	.	.	.
325	.	.	.	.	.	.
326	Resistant	Resistant	Sensetive	Resistant	Resistant	Sensetive
327	.	.	.	.	.	.
328	.	.	.	.	.	.
329	.	.	.	.	.	.
330	.	.	.	.	.	.
331	.	.	.	.	.	.
332	.	.	.	.	.	.
333	.	.	.	.	.	.
334	.	.	.	.	.	.
335	.	.	.	.	.	.
336	.	.	.	.	.	.
337	.	.	.	.	.	.
338	.	.	.	.	.	.
339	.	.	.	.	.	.
340	.	.	.	.	.	.
341	.	.	.	.	.	.
342	.	.	.	.	.	.
343	.	.	.	.	.	.
344	.	.	.	.	.	.
345	.	.	.	.	.	.
346	.	.	.	.	.	.
347	.	.	.	.	.	.
348	.	.	.	.	.	.
349	.	.	.	.	.	.
350	.	.	.	.	.	.

	NOR	CRO	AMC	CTX	CAZ	AMP
316	.	.	.	.	.	.
317	Sensetive	.	.	.	.	.
318	.	.	.	.	.	.
319	.	.	.	.	.	.
320	.	.	.	.	.	.
321	.	.	.	.	.	.
322	.	.	.	.	.	.
323	.	.	.	.	.	.
324	.	.	.	.	.	.
325	.	.	.	.	.	.
326	Sensetive	.	.	.	.	.
327	.	.	.	.	.	.
328	.	.	.	.	.	.
329	.	.	.	.	.	.
330	.	.	.	.	.	.
331	.	.	.	.	.	.
332	.	.	.	.	.	.
333	.	.	.	.	.	.
334	.	.	.	.	.	.
335	.	.	.	.	.	.
336	.	.	.	.	.	.
337	.	.	.	.	.	.
338	.	.	.	.	.	.
339	.	.	.	.	.	.
340	.	.	.	.	.	.
341	.	.	.	.	.	.
342	.	.	.	.	.	.
343	.	.	.	.	.	.
344	.	.	.	.	.	.
345	.	.	.	.	.	.
346	.	.	.	.	.	.
347	.	.	.	.	.	.
348	.	.	.	.	.	.
349	.	.	.	.	.	.
350	.	.	.	.	.	.

	AMK	GEN	ResPattern	MDR	GENTTWASH
316	.	.	.	.	After urination
317	.	.	R4	Yes	After defication
318	.	.	.	.	After defication
319	.	.	.	.	After defication
320	.	.	.	.	After urination
321	.	.	.	.	After urination
322	.	.	.	.	After urination
323	.	.	.	.	After urination
324	.	.	.	.	After defication
325	.	.	.	.	After defication
326	.	.	>=R5	Yes	After defication
327	.	.	.	.	After urination
328	.	.	.	.	After urination
329	.	.	.	.	Others
330	.	.	.	.	After urination
331	.	.	.	.	After urination
332	.	.	.	.	After urination
333	.	.	.	.	After defication
334	.	.	.	.	After urination
335	.	.	.	.	After urination
336	.	.	.	.	After urination
337	.	.	.	.	After urination
338	.	.	.	.	After urination
339	.	.	.	.	After defication
340	.	.	.	.	After urination
341	.	.	.	.	After urination
342	.	.	.	.	After defication
343	.	.	.	.	After urination
344	.	.	.	.	After urination
345	.	.	.	.	After urination
346	.	.	.	.	After urination
347	.	.	.	.	After urination
348	.	.	.	.	After urination
349	.	.	.	.	After defication
350	.	.	.	.	After urination

	ID	Age	AgeDscrptve	college	Residence	Department
351	32	23	21-25	Dessie coll...	Urban	Language
352	39	27	26-30	Dessie coll...	Urban	Language
353	26	21	21-25	Dessie coll...	Urban	Language
354	10	22	21-25	Dessie coll...	Urban	Language
355	27	22	21-25	Dessie coll...	Urban	Language
356	18	20	15-20	Dessie coll...	Urban	Language
357	43	20	15-20	Dessie coll...	Urban	Language
358	56	21	21-25	Dessie coll...	Urban	Language
359	32	20	15-20	Dessie coll...	Urban	Natural Sci...
360	21	20	15-20	Dessie coll...	Urban	Natural Sci...
361	35	20	15-20	Dessie coll...	Rural	Natural Sci...
362	38	20	15-20	Dessie coll...	Urban	Natural Sci...
363	53	23	21-25	Dessie coll...	Urban	Natural Sci...
364	41	19	15-20	Dessie coll...	Urban	Natural Sci...
365	68	22	21-25	Dessie coll...	Urban	Natural Sci...
366	73	20	15-20	Dessie coll...	Urban	Natural Sci...
367	92	19	15-20	Dessie coll...	Urban	Natural Sci...
368	55	20	15-20	Dessie coll...	Urban	Natural Sci...
369	28	20	15-20	Dessie coll...	Urban	Social Scie...
370	101	21	21-25	Dessie coll...	Urban	Social Scie...
371	16	22	21-25	Dessie coll...	Urban	Social Scie...
372	33	21	21-25	Dessie coll...	Urban	Natural Sci...
373	2	19	15-20	Dessie coll...	Urban	Language
374	11	23	21-25	Dessie coll...	Urban	Language
375	23	18	15-20	Dessie coll...	Urban	Language
376	34	22	21-25	Dessie coll...	Urban	Language
377	50	21	21-25	Dessie coll...	Urban	Language
378	2	22	21-25	Dessie coll...	Urban	Natural Sci...
379	8	22	21-25	Dessie coll...	Urban	Natural Sci...
380	16	20	15-20	Dessie coll...	Urban	Natural Sci...
381	19	23	21-25	Dessie coll...	Urban	Natural Sci...
382	21	20	15-20	Dessie coll...	Urban	Natural Sci...
383	13	29	26-30	Dessie coll...	Urban	Natural Sci...
384	27	19	15-20	Dessie coll...	Urban	Natural Sci...
385	31	29	26-30	Dessie coll...	Urban	Natural Sci...

	Batch	MartStatus	Income	Catheter	DM	HIV
351	Second Year	Single	>4000	No	No	No
352	Second Year	Single	1001-2000	No	No	No
353	Second Year	Single	>4000	No	No	No
354	Second Year	Single	≤1000	No	No	No
355	Second Year	Single	1001-2000	No	No	No
356	Second Year	Single	>4000	No	No	No
357	Second Year	Single	>4000	No	No	No
358	Second Year	Single	>4000	No	No	No
359	Second Year	Single	≤1000	No	No	No
360	Second Year	Single	1001-2000	No	No	No
361	Second Year	Single	≤1000	No	No	No
362	Second Year	Single	3001-4000	No	No	No
363	Second Year	Married	3001-4000	No	No	No
364	Second Year	Single	>4000	No	No	No
365	Second Year	Divorced	2001-3000	No	No	No
366	Second Year	Married	>4000	No	No	No
367	Second Year	Married	>4000	Yes	No	No
368	Second Year	Single	>4000	No	No	No
369	Second Year	Single	1001-2000	No	No	No
370	Second Year	Single	1001-2000	No	No	No
371	Second Year	Single	1001-2000	No	No	No
372	Second Year	Divorced	≤1000	No	No	No
373	Third Year	Single	2001-3000	No	No	Yes
374	Third Year	Single	≤1000	No	No	No
375	Third Year	Single	1001-2000	No	No	No
376	Third Year	Single	1001-2000	No	No	No
377	Third Year	Divorced	≤1000	No	No	No
378	Third Year	Single	>4000	No	No	No
379	Third Year	Divorced	>4000	No	No	No
380	Third Year	Single	>4000	No	No	No
381	Third Year	Married	1001-2000	No	No	No
382	Third Year	Married	1001-2000	No	No	No
383	Third Year	Married	>4000	No	No	No
384	Third Year	Married	>4000	No	No	No
385	Third Year	Single	≤1000	No	No	No

	STD	Hospitalization	Antibiotics	Contracepive	GentAbnorm	GentWash
351	No	No	No	Injection	No	During Urin...
352	No	No	No	No	No	during devi...
353	No	No	No	No	No	During Urin...
354	No	No	No	No	No	others
355	No	No	No	No	No	During Urin...
356	No	No	No	No	No	during devi...
357	No	No	No	No	No	During Urin...
358	No	No	No	Implant	No	every morni...
359	No	No	No	No	Yes	during devi...
360	No	No	No	Condom	No	during devi...
361	No	No	No	No	No	during devi...
362	No	Yes	Yes	No	No	during devi...
363	No	No	No	Implant	No	During Urin...
364	No	No	No	Condom	No	others
365	No	No	No	Implant	No	During Urin...
366	No	Yes	Yes	Injection	No	others
367	No	No	No	Pill	No	during devi...
368	No	No	No	Injection	No	others
369	No	No	No	Implant	No	during devi...
370	No	No	No	No	No	During Urin...
371	No	No	No	No	No	every morni...
372	No	No	No	Injection	No	every morni...
373	No	No	No	No	No	During Urin...
374	No	No	No	Injection	No	every morni...
375	No	No	No	No	No	During Urin...
376	No	No	No	No	No	During Urin...
377	No	No	No	Condom	No	every morni...
378	No	No	No	No	No	during devi...
379	No	No	No	Implant	No	others
380	No	No	No	No	No	During Urin...
381	No	No	No	Injection	No	others
382	No	No	No	Implant	No	During Urin...
383	No	No	No	Condom	No	During Urin...
384	No	No	No	Implant	Yes	During Urin...
385	No	No	No	IUD	No	During Urin...

	SexFrequency	SU	BactSpp	FungSpp	CL	E
351	<3 times	Negative	.	.	.	.
352	NO	Postive	E.coli	Candidia tr...	.	.
353	NO	Postive	.	Candidia tr...	.	.
354	NO	Negative	.	.	.	.
355	NO	Negative	.	.	.	.
356	NO	Postive	.	Candidia tr...	.	.
357	NO	Postive	.	Candidia tr...	.	.
358	<3 times	Postive	S. saproph...	.	Sensetive	Intermidate
359	NO	Postive	S. saproph...	.	Intermideate	Intermidate
360	NO	Postive	.	Candidia al...	.	.
361	<3 times	Postive	Klebsella o...	.	.	.
362	NO	Negative	.	.	.	.
363	NO	Negative	.	.	.	.
364	NO	Negative	.	.	.	.
365	NO	Negative	.	.	.	.
366	>=3 times	Negative	.	.	.	.
367	NO	Negative	.	.	.	.
368	NO	Negative	.	.	.	.
369	<3 times	Negative	.	.	.	.
370	NO	Negative	.	.	.	.
371	NO	Negative	.	.	.	.
372	<3 times	Negative	.	.	.	.
373	NO	Negative	.	.	.	.
374	<3 times	Negative	.	.	.	.
375	NO	Negative	.	.	.	.
376	NO	Negative	.	.	.	.
377	<3 times	Postive	E.coli	.	.	.
378	NO	Negative	.	.	.	.
379	NO	Negative	.	.	.	.
380	NO	Negative	.	.	.	.
381	NO	Negative	.	.	.	.
382	<3 times	Negative	.	.	.	.
383	NO	Negative	.	.	.	.
384	<3 times	Negative	.	.	.	.
385	NO	Postive	Providencia...	.	.	.

	PEN	CAF	CIP	TTC	SXT	F
351	.	.	.	.	.	.
352	.	.	Sensetive	Resistant	Resistant	Sensetive
353	.	.	.	.	.	.
354	.	.	.	.	.	.
355	.	.	.	.	.	.
356	.	.	.	.	.	.
357	.	.	.	.	.	.
358	Resistant	Resistant	Intermidate	Resistant	Resistant	Sensetive
359	Resistant	Resistant	Sensetive	Resistant	Resistant	Sensetive
360	.	.	.	.	.	.
361	.	.	Sensetive	Resistant	Resistant	Resistant
362	.	.	.	.	.	.
363	.	.	.	.	.	.
364	.	.	.	.	.	.
365	.	.	.	.	.	.
366	.	.	.	.	.	.
367	.	.	.	.	.	.
368	.	.	.	.	.	.
369	.	.	.	.	.	.
370	.	.	.	.	.	.
371	.	.	.	.	.	.
372	.	.	.	.	.	.
373	.	.	.	.	.	.
374	.	.	.	.	.	.
375	.	.	.	.	.	.
376	.	.	.	.	.	.
377	.	.	Resistant	Resistant	Sensetive	Sensetive
378	.	.	.	.	.	.
379	.	.	.	.	.	.
380	.	.	.	.	.	.
381	.	.	.	.	.	.
382	.	.	.	.	.	.
383	.	.	.	.	.	.
384	.	.	.	.	.	.
385	.	.	Sensetive	.	Resistant	Sensetive

spss data Bre.sav

	NOR	CRO	AMC	CTX	CAZ	AMP
351	.	.	.	.	.	.
352	Sensetive	Sensetive	Resistant	Sensetive	Intermidate	Resistant
353	.	.	.	.	.	.
354	.	.	.	.	.	.
355	.	.	.	.	.	.
356	.	.	.	.	.	.
357	.	.	.	.	.	.
358	Sensetive	.	.	.	.	.
359	Sensetive	.	.	.	.	.
360	.	.	.	.	.	.
361	Sensetive	Intermidiate	Resistant	Intermidate	Intermidate	.
362	.	.	.	.	.	.
363	.	.	.	.	.	.
364	.	.	.	.	.	.
365	.	.	.	.	.	.
366	.	.	.	.	.	.
367	.	.	.	.	.	.
368	.	.	.	.	.	.
369	.	.	.	.	.	.
370	.	.	.	.	.	.
371	.	.	.	.	.	.
372	.	.	.	.	.	.
373	.	.	.	.	.	.
374	.	.	.	.	.	.
375	.	.	.	.	.	.
376	.	.	.	.	.	.
377	Sensetive	Sensetive	Resistant	Sensetive	Sensetive	Resistant
378	.	.	.	.	.	.
379	.	.	.	.	.	.
380	.	.	.	.	.	.
381	.	.	.	.	.	.
382	.	.	.	.	.	.
383	.	.	.	.	.	.
384	.	.	.	.	.	.
385	Sensetive	Intermidiate	Resistant	Intermidate	Resistant	Resistant

	AMK	GEN	ResPattern	MDR	GENTTWASH
351	.	.	.	.	Afterurination
352	Resistant	Resistant	>=R5	Yes	Afterdefication
353	.	.	.	.	Afterurination
354	.	.	.	.	Afterurination
355	.	.	.	.	Afterurination
356	.	.	.	.	Afterurination
357	.	.	.	.	Afterurination
358	.	.	>=R5	Yes	Afterurination
359	.	.	>=R5	Yes	Afterdefication
360	.	.	.	.	Afterdefication
361	Intermidiate	Sensetive	>=R5	Yes	Afterdefication
362	.	.	.	.	Afterdefication
363	.	.	.	.	Afterurination
364	.	.	.	.	Others
365	.	.	.	.	Afterurination
366	.	.	.	.	Others
367	.	.	.	.	Afterdefication
368	.	.	.	.	Afterdefication
369	.	.	.	.	Afterdefication
370	.	.	.	.	Afterurination
371	.	.	.	.	Everymorning
372	.	.	.	.	Everymorning
373	.	.	.	.	Afterurination
374	.	.	.	.	Everymorning
375	.	.	.	.	Afterurination
376	.	.	.	.	Afterurination
377	Sensetive	Sensetive	R4	Yes	Everymorning
378	.	.	.	.	Afterdefication
379	.	.	.	.	Afterurination
380	.	.	.	.	Afterurination
381	.	.	.	.	Afterurination
382	.	.	.	.	Afterurination
383	.	.	.	.	Afterurination
384	.	.	.	.	Afterurination
385	Resistant	.	>=R5	Yes	Afterurination

	ID	Age	AgeDscrptve	college	Residence	Department
386	38	19	15-20	Dessie coll...	Urban	Natural Sci...
387	43	20	15-20	Dessie coll...	Urban	Natural Sci...
388	52	19	15-20	Dessie coll...	Rural	Natural Sci...
389	42	21	21-25	Dessie coll...	Urban	Natural Sci...
390	60	19	15-20	Dessie coll...	Urban	Natural Sci...
391	63	20	15-20	Dessie coll...	Rural	Natural Sci...
392	69	19	15-20	Dessie coll...	Urban	Natural Sci...
393	71	21	21-25	Dessie coll...	Urban	Natural Sci...
394	76	20	15-20	Dessie coll...	Rural	Natural Sci...
395	82	23	21-25	Dessie coll...	Rural	Natural Sci...
396	86	22	21-25	Dessie coll...	Urban	Natural Sci...
397	93	20	15-20	Dessie coll...	Rural	Natural Sci...
398	99	20	15-20	Dessie coll...	Urban	Natural Sci...
399	75	23	21-25	Dessie coll...	Urban	Social Scie...
400	15	23	21-25	Dessie coll...	Urban	Social Scie...
401	5	20	15-20	Dessie Hea...	Rural	Social Scie...
402	13	21	21-25	Dessie coll...	Rural	Natural Sci...
403	21	20	15-20	Dessie coll...	Urban	Natural Sci...
404	33	22	21-25	Dessie coll...	Urban	Natural Sci...
405	28	22	21-25	Dessie coll...	Urban	Natural Sci...
406	111	23	21-25	Alkan Healt...	Urban	Health Scie...
407	116	24	21-25	Alkan Healt...	Urban	Health Scie...
408	132	22	21-25	Alkan Healt...	Urban	Health Scie...
409	123	26	26-30	Alkan Healt...	Urban	Health Scie...
410	100	23	21-25	Alkan Healt...	Urban	Health Scie...
411	1	21	21-25	Alkan Healt...	Urban	Health Scie...
412	7	26	26-30	Alkan Healt...	Urban	Health Scie...
413	13	22	21-25	Alkan Healt...	Urban	Health Scie...
414	27	23	21-25	Alkan Healt...	Urban	Health Scie...
415	31	21	21-25	Alkan Healt...	Urban	Health Scie...
416	43	21	21-25	Alkan Healt...	Urban	Health Scie...
417	19	21	21-25	Alkan Healt...	Urban	Health Scie...
418	44	22	21-25	Alkan Healt...	Urban	Health Scie...
419	53	24	21-25	Alkan Healt...	Urban	Health Scie...
420	68	21	21-25	Alkan Healt...	Urban	Health Scie...

	Batch	MartStatus	Income	Catheter	DM	HIV
386	Third Year	Single	3001-4000	No	No	No
387	Third Year	Single	>4000	No	No	No
388	Third Year	Single	3001-4000	No	No	No
389	Third Year	Single	>4000	No	No	No
390	Third Year	Single	3001-4000	No	No	No
391	Third Year	Single	3001-4000	No	No	No
392	Third Year	Single	1001-2000	No	No	No
393	Third Year	Divorced	≤1000	No	No	No
394	Third Year	Single	1001-2000	No	No	No
395	Third Year	Single	≤1000	No	No	No
396	Third Year	Single	1001-2000	No	No	No
397	Third Year	Single	2001-3000	No	No	No
398	Third Year	Single	>4000	Yes	No	No
399	Third Year	Single	>4000	No	No	No
400	Third Year	Married	3001-4000	Yes	No	No
401	Third Year	Single	2001-3000	No	No	No
402	Third Year	Single	>4000	No	No	No
403	Third Year	Single	1001-2000	No	No	No
404	Third Year	Married	1001-2000	No	No	No
405	Third Year	Single	1001-2000	No	No	No
406	Fourth Year	Married	>4000	No	Yes	No
407	Fourth Year	Single	1001-2000	No	No	No
408	Fourth Year	Single	>4000	No	No	No
409	Fourth Year	Single	>4000	No	No	No
410	Fourth Year	Single	>4000	No	No	No
411	Third Year	Single	>4000	No	No	No
412	Third Year	Single	>4000	No	No	No
413	Third Year	Single	>4000	No	No	No
414	Third Year	Single	>4000	No	No	No
415	Third Year	Single	3001-4000	No	No	No
416	Third Year	Single	>4000	No	No	No
417	Third Year	Single	3001-4000	No	No	No
418	Third Year	Single	>4000	No	No	No
419	Third Year	Single	>4000	No	No	No
420	Third Year	Single	>4000	Yes	No	No

	STD	Hospitalization	Antibiotics	Contracepive	GentAbnorm	GentWash
386	No	No	No	Injection	No	every morni...
387	No	No	No	No	No	During Urin...
388	No	No	No	No	No	During Urin...
389	No	No	No	Implant	No	During Urin...
390	No	Yes	Yes	No	No	During Urin...
391	No	No	No	No	No	others
392	No	No	No	Implant	No	during devi...
393	No	No	No	Condom	No	others
394	No	No	No	Injection	No	during devi...
395	No	No	No	No	No	others
396	No	No	No	No	No	during devi...
397	No	No	No	Condom	No	During Urin...
398	No	No	No	No	No	During Urin...
399	No	No	No	No	No	During Urin...
400	No	No	No	Implant	No	during devi...
401	No	No	No	Implant	No	during devi...
402	No	No	No	No	No	during devi...
403	No	No	No	No	No	others
404	No	No	No	Pill	No	During Urin...
405	No	No	No	No	No	during devi...
406	No	No	No	Pill	No	During Urin...
407	No	No	No	Condom	No	During Urin...
408	Yes	No	No	No	Yes	others
409	No	No	No	No	No	During Urin...
410	No	No	No	Pill	No	during devi...
411	No	No	No	No	No	During Urin...
412	No	No	No	No	No	During Urin...
413	No	No	No	No	No	During Urin...
414	No	No	No	No	No	others
415	No	No	No	Condom	No	During Urin...
416	No	No	No	No	No	During Urin...
417	No	No	No	Pill	No	during devi...
418	No	No	No	No	No	During Urin...
419	No	Yes	Yes	Implant	No	every morni...
420	No	No	No	No	No	During Urin...

	SexFrequency	SU	BactSpp	FungSpp	CL	E
386	<3 times	Postive	S. saproph...	Candidia al...	Sensetive	Intermidate
387	NO	Negative	.	.	.	.
388	NO	Negative	.	.	.	.
389	<3 times	Negative	.	.	.	.
390	NO	Negative	.	.	.	.
391	NO	Negative	.	.	.	.
392	NO	Negative	.	.	.	.
393	NO	Negative	.	.	.	.
394	NO	Negative	.	.	.	.
395	NO	Negative	.	.	.	.
396	NO	Negative	.	.	.	.
397	NO	Negative	.	.	.	.
398	NO	Negative	.	.	.	.
399	NO	Negative	.	.	.	.
400	<3 times	Postive	E.coli	.	.	.
401	NO	Negative	.	.	.	.
402	NO	Postive	.	Candidia gl...	.	.
403	NO	Negative	.	.	.	.
404	NO	Negative	.	.	.	.
405	NO	Negative	.	.	.	.
406	NO	Negative	.	.	.	.
407	NO	Negative	.	.	.	.
408	NO	Postive	S. saproph...	.	Sensetive	Intermidate
409	NO	Postive	.	Candidia tr...	.	.
410	>=3 times	Postive	S. saproph...	.	Sensetive	Sensetive
411	NO	Postive	.	Candidia kr...	.	.
412	NO	Negative	.	.	.	.
413	NO	Negative	.	.	.	.
414	NO	Negative	.	.	.	.
415	NO	Negative	.	.	.	.
416	NO	Negative	.	.	.	.
417	<3 times	Negative	.	.	.	.
418	NO	Negative	.	.	.	.
419	<3 times	Postive	serratia	.	.	.
420	NO	Postive	.	.	.	.

	PEN	CAF	CIP	TTC	SXT	F
386	Resistant	Resistant	Sensetive	Sensetive	Resistant	Sensetive
387	.	.	.	.	.	.
388	.	.	.	.	.	.
389	.	.	.	.	.	.
390	.	.	.	.	.	.
391	.	.	.	.	.	.
392	.	.	.	.	.	.
393	.	.	.	.	.	.
394	.	.	.	.	.	.
395	.	.	.	.	.	.
396	.	.	.	.	.	.
397	.	.	.	.	.	.
398	.	.	.	.	.	.
399	.	.	.	.	.	.
400	.	.	Sensetive	Resistant	Sensetive	Sensetive
401	.	.	.	.	.	.
402	.	.	.	.	.	.
403	.	.	.	.	.	.
404	.	.	.	.	.	.
405	.	.	.	.	.	.
406	.	.	.	.	.	.
407	.	.	.	.	.	.
408	Resistant	Resistant	Sensetive	Resistant	Resistant	Sensetive
409	.	.	.	.	.	.
410	Resistant	Resistant	Sensetive	Intermidate	Resistant	Sensetive
411	.	.	.	.	.	.
412	.	.	.	.	.	.
413	.	.	.	.	.	.
414	.	.	.	.	.	.
415	.	.	.	.	.	.
416	.	.	.	.	.	.
417	.	.	.	.	.	.
418	.	.	.	.	.	.
419	.	.	Resistant	.	Intermidiate	Sensetive
420	.	.	.	.	.	.

	NOR	CRO	AMC	CTX	CAZ	AMP
386	Sensetive	.	.	.	.	.
387	.	.	.	.	.	.
388	.	.	.	.	.	.
389	.	.	.	.	.	.
390	.	.	.	.	.	.
391	.	.	.	.	.	.
392	.	.	.	.	.	.
393	.	.	.	.	.	.
394	.	.	.	.	.	.
395	.	.	.	.	.	.
396	.	.	.	.	.	.
397	.	.	.	.	.	.
398	.	.	.	.	.	.
399	.	.	.	.	.	.
400	Sensetive	Sensetive	Resistant	Sensetive	Sensetive	Resistant
401	.	.	.	.	.	.
402	.	.	.	.	.	.
403	.	.	.	.	.	.
404	.	.	.	.	.	.
405	.	.	.	.	.	.
406	.	.	.	.	.	.
407	.	.	.	.	.	.
408	Sensetive	.	.	.	.	.
409	.	.	.	.	.	.
410	Sensetive	.	.	.	.	.
411	.	.	.	.	.	.
412	.	.	.	.	.	.
413	.	.	.	.	.	.
414	.	.	.	.	.	.
415	.	.	.	.	.	.
416	.	.	.	.	.	.
417	.	.	.	.	.	.
418	.	.	.	.	.	.
419	Sensetive	Sensetive	.	Sensetive	Resistant	Resistant
420	.	.	.	.	.	.

	AMK	GEN	ResPattern	MDR	GENTTWASH
386	.	.	R4	Yes	Everymorning
387	.	.	.	.	Afterurination
388	.	.	.	.	Afterurination
389	.	.	.	.	Afterurination
390	.	.	.	.	Afterurination
391	.	.	.	.	Afterdefication
392	.	.	.	.	Afterdefication
393	.	.	.	.	Afterurination
394	.	.	.	.	Afterdefication
395	.	.	.	.	Afterdefication
396	.	.	.	.	Afterdefication
397	.	.	.	.	Afterurination
398	.	.	.	.	Afterurination
399	.	.	.	.	Afterurination
400	Resistant	Sensetive	R4	Yes	Afterdefication
401	.	.	.	.	Afterdefication
402	.	.	.	.	Afterdefication
403	.	.	.	.	Afterdefication
404	.	.	.	.	Afterurination
405	.	.	.	.	Afterdefication
406	.	.	.	.	Afterurination
407	.	.	.	.	Afterurination
408	.	.	>=R5	Yes	Others
409	.	.	.	.	Afterurination
410	.	.	R4	Yes	Afterdefication
411	.	.	.	.	Afterurination
412	.	.	.	.	Afterurination
413	.	.	.	.	Afterurination
414	.	.	.	.	Afterurination
415	.	.	.	.	Afterurination
416	.	.	.	.	Afterurination
417	.	.	.	.	Afterdefication
418	.	.	.	.	Afterurination
419	Sensetive	Sensetive	R4	Yes	Everymorning
420	.	.	.	.	Afterurination

	ID	Age	AgeDscrptve	college	Residence	Department
421	76	23	21-25	Alkan Healt...	Urban	Health Scie...
422	23	21	21-25	Alkan Healt...	Urban	Health Scie...
423	34	22	21-25	Alkan Healt...	Urban	Health Scie...

	Batch	MartStatus	Income	Catheter	DM	HIV
421	Third Year	Single	>4000	No	No	No
422	Third Year	Single	>4000	No	No	No
423	Third Year	Single	2001-3000	No	No	No

	STD	Hospitalization	Antibiotics	Contracepive	GentAbnorm	GentWash
421	No	No	No	No	No	others
422	No	No	No	No	No	During Urin...
423	No	No	No	No	No	others

spss data Bre.sav

	SexFrequency	SU	BactSpp	FungSpp	CL	E
421	NO	Negative	.	.	.	.
422	<3 times	Negative	.	.	.	.
423	NO	Negative	.	.	.	.

	PEN	CAF	CIP	TTC	SXT	F
421	.	.	.	.	.	.
422	.	.	.	.	.	.
423	.	.	.	.	.	.

	NOR	CRO	AMC	CTX	CAZ	AMP
421	.	.	.	.	.	.
422	.	.	.	.	.	.
423	.	.	.	.	.	.

	AMK	GEN	ResPattern	MDR	GENTTWASH
421	.	.	.	.	After urination
422	.	.	.	.	After urination
423	.	.	.	.	After defication
