# Supplementary material for: Antibiogram of uropathogens and associated risk factors among asymptomatic female college students in Dessie town, Northeast Ethiopia
Source: PLoS One. 2023 Nov 29;18(11):e0276033. doi: 10.1371/journal.pone.0276033 (PMC10686425; doi:10.1371/journal.pone.0276033)
Supplement: Attachment — Submitted filename: Reviewers comment for Antibiogram manuscript_110123.pdf [file pone.0276033.s003.pdf]

**Manuscript title**: **Magnitude and antibiogram of uropathogens and associated risk factors among asymptomatic female college students in Dessie town, Northeast Ethiopia**

Thank you, the Editor PLOS ONE Global Public Health, for the invite to review the above-named manuscript. The authors research topic is public health significance with the perspective of the laboratory component of the study.

Please see below comments for the kind consideration of the authors aimed at adding value to the research work. Thank you.

**General comment**

1. The line numbering was not consistent across the body of the manuscript text. Authors need to ensure proper line numbering.

**Abstract**

1. Objectives: The objectives are captured as if the study is yet to be done. Authors need to frame the objectives in past tense.
2. Methods: p value supposed to be $<0.05$ and not $\leq 0.05$
3. Result: Authors should maintain 1 decimal place for the proportion, and 2 decimal places for the Adjusted odds ratio and 95% confidence interval. P-value should also be in 2 decimal places.

**Materials and methods**

Line number 15: Suggested sequence to follow as follows: Study area, Study design, Study population, Eligibility criteria (Inclusion and Exclusion criteria), Sample size determination, Sampling technique, Data collection method(s) and Data analysis.

Line numbers 17-25: Need for authors to reframe the methods section.

Line numbers 19-22: Authors need to provide detailed information on how the study areas were selected.

Line number 23: This is the sample size, authors need to provide detailed information on how the sample size calculation was done.

Line number 22: ‘’female college students’’: These are study population. There is need for authors to incorporate eligibility criteria (inclusion and exclusion criteria)

Line numbers 23-25: Authors should provide detailed information on the sampling technique used to select eligible respondents for interview

Line number 27: Authors to state the method of questionnaire administration: self or interviewer administered?

Line number 28: Clinical and risk factors data on ……….? Need for the authors to provide the need information.

Line number 5 under specimen transportation: ‘’status’’ to be added after non-pregnancy

Line 22 under Cultivation and identification of isolates: was changed to ‘’were’’

Line 28 under Cultivation and identification of isolates: sub cultured changed to subcultured

Lines 13-20 under Antimicrobial Susceptibility: Drug names to start with Capital letter

Line 25 under Antimicrobial Susceptibility: discs ‘’was’’ measured ……. Change ‘’was’’ to ‘’were’’

Line 26 under Antimicrobial Susceptibility: susceptibility tests ‘’was’’ based …… Change ‘’was’’ to ‘’were’’

Line 3 under Quality assurance: questionnaires ‘’was’’ checked by … Change ‘’was’’ to ‘’were’’

Lines 3-5: ‘’The questionnaire was pretested on 5%.........., Northeast Ethiopia. It will be good if the authors could provide information on feedback from the pre-test and it helped on improving the quality of the study.

Lines 21-22: ‘’To show the relationship between each variable……was utilized’’. Test statistic to assess association between dependent and independent variables is Odds ratio (Crude OR)

(with 95% CI) OR Chi square (with p-value). This is bivariate analysis. Binary logistic regression is a multivariate analysis used to assess predictors of a dependent or outcome variable. Authors should also provide information on Dependent (outcome) and Independent (explanatory) variables. The sequence supposed to be univariate, bivariate, and multivariate analysis.

Line 25: Ethical approval and consent to participate: For journal publication, authors should situate the ethical considerations sub-section based on the journal's guidelines.

**Results**

Line 5: Authors should include the response rate based on the number of eligible respondents reached.

Line 9: Table 1 to be included after students

Line number 14 ‘’15 independent variables’’; Authors should provide information on the independent variables under the methods section

Authors should maintain 2 decimal points for information on results section.

Line 16: AOR: Adjusted Odds Ratio. This should be mentioned under the methods section and written in full with the binary logistic regression done.

Line 20: Authors should use greater than or equal to sign

Line 21: (47.9) and (13.8), Authors to insert % to 47.9 and 13.8

Line 3 under Multiple drug resistance patterns of the isolates: This should be defined under the methods section. Need for authors to include ''Operational definitions'' under the methods section.

**Discussion**

The first paragraph of the Discussion should first provide information on summary of the study based on the study objectives. This is important to provide information if the study objectives is achieved or not.

Line 11 and 18: The use of the ''i.e'' acronym should be deleted.

Lines 15-16: ''This is in agreement with studies in Nigeria'' This is incomplete. Need for authors to provide information on the type of study conducted.

Line 17: relatively higher than the findings in Ghana: Need to elaborate on the study information in Ghana.

Line 20: ''findings was lower….from Nigeria'': Authors should be explicit on the type of information in relation to Nigeria.

Line 24 and 25: ''Furthermore, bacteriuria among pediatircs peak during ………………'' Reference to be provided.

Line 20-23: ''Our finding revealed that the frequency of sexual intercourse………: This is repetition of results, authors are advised to delete.

Lines 26-28: ''According to our investigation, prevalence of UTI……..'' This is repetition of results, authors are advised to delete.

Line 8-11: '' In the present study, there was no statistically significant association between prevalence of UTI in asymptomatic female college students and age, residence (colleges), student batch, monthly family income, marital status, history of UTI, history of catheterization, history of STDs, and presence of genitourinary abnormalities. Similar finding was reported from Nigeria''. I do not think this statement is important.

Line 1: According to the international standard for definition of drug resistance ''This information supposed to be part of the operational definitions under the methods section''.

**Limitations**

Lines 22 and 23: Study limitations are factors which could affect the outcome of the study but however, measures were taken in addressing or mitigating such. The information provided by the authors is not a study limitation.

Line 24: List of abbreviations: Authors should ensure the list of abbreviations should be captured the way they appear in the body of the manuscript.

Figure 1: The title should be underneath the chart.

Table 1: Unequal class width for respondents age. Authors should ensure class width are equal. What does ETB means? This supposed to be captured under the abbreviation section.

Table 2: Authors should maintain 2 decimal points all through.

Others: under genital area washing habit include the females who wash their vaginal area 5-7 times per day. **SUP**

= Significant Uropathogen, **AOR** = adjusted odds ratio, **COR** = crude odds ratio, **1** = reference group, **95% CI** = 95% confidence interval, **DM** = Diabetic mellitus, **UTI** = urinary tract infection, **NA** = not applicable. ''This is to be included in the abbreviation section.

Table 3: Frequency of bacterial and fungal uropathogens of asymptomatic UTI among female college students in Dessie town, Northeast Ethiopia, January 2021 - March 2021.: Authors to include N=104 with the title. ''from 104'' to be deleted from N (%)

Antimicrobial resistance pattern (R0-R5) in Table 6: The antimicrobial resistance pattern supposed to be mentioned under the operational definitions of the methods section.
