# Supplementary material for: Antibiogram of uropathogens and associated risk factors among asymptomatic female college students in Dessie town, Northeast Ethiopia
Source: PLoS One. 2023 Nov 29;18(11):e0276033. doi: 10.1371/journal.pone.0276033 (PMC10686425; doi:10.1371/journal.pone.0276033)
Supplement: Attachment — Submitted filename: Comments PONE-D-22-26752.docx [file pone.0276033.s002.docx]

**Comments**

**I found the manuscript very interesting because of ignored population but need improvement regarding conceptualization, language used in it ( English, consult with native)**

1. **Get through the whole Abstract need improvement in language**
2. **In line 4-6 authors use old reference, the ref should be updated of recent couple of years (update references in the whole manuscript if possible especially in introduction and discussion).**
3. **The sentence used in ref 3 is not strong statement, rephrase it.**
4. **From line 27 to ref 14, statements are not clear having grammatical mistakes, kindly correct it and also use new refs.**
5. **In discussion portion, in line 9-14 language typos are found kindly correct it.**
6. **In line 17-20 (Conclusion portion) the future prospective is not clear.**
