# Supplementary material for: Antibiogram of uropathogens and associated risk factors among asymptomatic female college students in Dessie town, Northeast Ethiopia
Source: PLoS One. 2023 Nov 29;18(11):e0276033. doi: 10.1371/journal.pone.0276033 (PMC10686425; doi:10.1371/journal.pone.0276033)
Supplement: Attachment — Submitted filename: Response to Reviewers.docx [file pone.0276033.s004.docx]

**Author’s Response to Editor and Reviewer comments**

**Manuscript ID number:**
PONE-D-22-26752

**Title of paper:**
Magnitude and antibiogram of uropathogens and associated risk factors among asymptomatic female college students in Dessie town, Northeast Ethiopia

**General**

We thank all the reviewers for critically reviewing our manuscript which helps us to render better clarity to the paper and make it scientifically plausible. We thank the PLOS ONE academic editors for their valuable comments and for giving us an opportunity to revise the manuscript.

All the questions raised by the Reviewers and Editor have been addressed; and the manuscript is modified accordingly. Moreover, all the requested Editorial corrections are addressed in both the revised manuscript and the response letter. We have also updated our Data availability statements as “all available data were included in the manuscript”. Please update the Data Availability statement to reflect the information we provide. Changes are shown with track changes in the file labeled ‘Revised Manuscript with Track Changes’. The point-by-point response is given below.

**Editor Comments:**

1. Please ensure that your manuscript meets PLOS ONE's style requirements, including those for file naming.

***Author’s Response:***

- Thanks for the significant editorial comment. The revised manuscript is now updated and meets PLOS ONE's style requirements, including those for file naming.

2. We note that you have stated that you will provide repository information for your data at acceptance. Should your manuscript be accepted for publication, we will hold it until you provide the relevant accession numbers or DOIs necessary to access your data. If you wish to make changes to your Data Availability statement, please describe these changes in your cover letter and we will update your Data Availability statement to reflect the information you provide.

***Author’s Response:***

- We didn’t have data repository. We make changes to our Data Availability Statement as all available data were included in the manuscript. We also state the changes in the cover letter.

3. Please include your tables as part of your main manuscript and remove the individual files. Please note that supplementary tables (should remain/ be uploaded) as separate ""supporting information"" files. Please review your reference list to ensure that it is complete and correct.

***Author’s Response:***

- Thank you and we include the tables as part of the main manuscript in our revision. We have also reviewed our list of references making sure they are complete and correct.

**Reviewer #1 comments**

1. Get through the whole Abstract need improvement in language.

***Author’s Response:***

- Thanks for your comment and we made the necessary amendments on the language errors. We have done language editing by a native expert.

2. In line 4-6 authors use old reference, the ref should be updated of recent couple of years (update references in the whole manuscript if possible, especially in introduction and discussion).

***Author’s Response:***

- Thank you for the substantial comment. We have incorporated a recent reference as per the comment, and statements are updated accordingly. This new reference is added in place of an old reference #2: Luu T, Albarillo FS. Asymptomatic Bacteriuria: Prevalence, Diagnosis, Management, and Current Antimicrobial Stewardship Implementations. Am J Med. 2022; 135(8):e236-e244.

3. The sentence used in ref 3 is not strong statement, rephrase it.

***Author’s Response:***

- Comment well taken and statement is rephrased.

4. From line 27 to ref 14, statements are not clear having grammatical mistakes, kindly correct it and also use new refs.

***Author’s Response:***

- Thank you for the valuable comment. We correct statements with grammatical mistakes, and replace old reference (ref #9) with new one: Storme O, Tirán Saucedo J, Garcia-Mora A, Dehesa-Dávila M, Naber KG. Risk factors and predisposing conditions for urinary tract infection. Ther Adv Urol. 2019; 2(11).

5. In discussion portion, in line 9-14 language typos are found kindly correct it.

***Author’s Response:***

- Thank you and corrections are made on language typos.

6. In line 17-20 (Conclusion portion) the future prospective is not clear.

***Author’s Response:***

- Comment accepted and clear future perspective is added to the end of the conclusion section.

**Reviewer #2 comments**

1. Title: I suggest the title - Antibiogram of Uropathogens and associated risk factors among asymptomatic female college students in Dessie town, Northeast Ethiopia. Delete “Magnitude and” Please feel free to amend.

Short title: Same thing Delete “Magnitude

***Author’s Response:***

- Thanks for your constructive comment and we have made the necessary amendments.

2. ABSTRACT:

Line 1- Delete “Asymptomatic” in the bracket and replace with “asymptomatic”

Line 7- Delete “asymptomatic”. It is repeated twice in the sentence. The sentence can read “to determine the prevalence and factors associated with urinary tract infection, and antibiogram of the uropathogen isolates among asymptomatic female college students”

Line 9 – Delete “found”

***Author’s Response:***

- Thanks for your constructive comment and suggestion. Based on your suggestion we have made the necessarily change on the title of the manuscript.

3. Line 10- Do you have any listed criteria for inclusion and exclusion of participants? Also, what do you mean by "reproductive age"? Can you explain by including or representing this with an age group or age range?

***Author’s Response:***

- Thank you for the question. We included study participants who were non-pregnant female college students without symptoms of UTI, and we have also checked their willingness to participate in this study. Moreover, the inclusion and exclusion criteria are clearly mentioned in the method section of the abstract and the body of the manuscript. Regarding “reproductive age” we have used the WHO age category 15-49 years as reproductive age. We have included this age group in the required area of the manuscript.

4. Lines 13-16- This can read as follows for clarity “Data were coded and entered for statistical analysis using SPSS version 22.0. Descriptive statistics, bivariate and multivariate logistic regression analysis were performed and p-values ≤0.05 with corresponding 95% confidence interval (CI) were considered statistically significant”

Line 19 - What does S mean? Provide the full name of pathogen before abbreviation.

***Author’s Response:***

- Thank you for your helpful comment and suggestion, and we have made the necessarily modification.

5. Line 24- please put commas in between the drugs – “Gram positive bacterial isolates were sensitive to norfloxacin, clindamycin, and ciprofloxacin accounting for 24 (82.7%), 20 (69.0%), and 19 (65.5%), respectively.

***Author’s Response:***

- Thank you for your practical comment and as per your comment, we have used commas between the drugs in this specific sentence.

6. Lines 28-32 – please these lines are unclear. Rephrase for clarity. You may want to start by saying “Factors identified to be associated with UTI in asymptomatic female college students included frequency of sexual intercourse --------------“what do you mean by every morning? Early morning what?

***Author’s Response:***

- Comment well taken and statement is rephrased. A significant number of participants mentioned that they wash their genital area only every morning before going to class. This genital area washing habit is quite common in Ethiopia, especially in the rural area, where females couldn’t have access to water due to the unavoidable open defecation habits. Thus, college students who came from the rural area continue this habit despite they reside in urban city.

7. Lines 34-34- regular health education on the risk of asymptomatic infectious diseases for who?

***Author’s Response:***

- Thank you for your constructive comment. We planned to provide this health education for female population who are at the reproductive age group. We have included this in the abstract section and in the conclusion part of the manuscript.

8. INTRODUCTION:

Line 7- delete “in a community”

Line 11- add “to be” affected

Lines 14-15 – Please rephrase the sentence or delete “while in symptomatic, signs are the major one”.

***Author’s Response:***

- Comment accepted and amendments are made.

9. Line 18 – add full stop at the end of the sentence,

Line 19 “delete “ASB” and stick to UTI

Line 23: delete “the most”

***Author’s Response:***

- Thank you for the valuable input. Modifications are made accordingly.

10. Page 2 lines 5-6 not clear- please rephrase

***Author’s Response:***

- Comments well taken and the manuscript corrected accordingly, thanks.

11. MATERIALS AND METODS:

Line 17- from January 2021 - March 2021 should end the sentence

***Author’s Response:***

- Comments taken and the manuscript corrected accordingly, thanks.

12. Line 18-19- how was this done? Please provide details”. Also provide details of how participants were systematically selected.

Please provide a section on how the sample size for this study was estimated.

***Author’s Response:***

- Thank you and details of participant selection is provided in a new section named ‘sample size determination and sampling technique’.

13. Provide your inclusion and exclusion criteria for selection of participants.

***Author’s Response:***

- Comments taken and inclusion and exclusion criteria are provided accordingly, thanks.

14. Please provide a reference number for the ethical approval

***Author’s Response:***

- Thank you and reference number (CMHS-210/13/13) was provided for the ethical approval in the ethical consideration section of the manuscript.

15. Data and specimen collection:

Line 27- provide details of how the questionnaire was designed and structured? Were they personally administered or not? What demographic, clinical, and risk factors data were collected? Are questions contingency, matrix, open or closed ended questions? Was this tool pretested? How?

***Author’s Response:***

- Thank you and all issues are addressed in the revised manuscript.

16. Line 28- change to “10 mL”. Please effect changes to the unit throughout the document

Pg 3 Line 14- CFU/ml change to “CFU/mL”. Effect changes throughout the document.

***Author’s Response:***

- Thank you and all issues are addressed in the revised manuscript.

17. Pg 4 Line 2- standard clinical laboratory methods (how? please be detailed)

***Author’s Response:***

- Thank you for the critical comment. We have used phenotypic detection methods for the uropathogens. And this is mentioned in the revised manuscript together with an appropriate citation.

18. Line 24- ”as a resistant” delete

***Author’s Response:***

- Comment accepted and corrections made accordingly.

19. Line 29- What are these quality control tests performed. Please describe.

***Author’s Response:***

- Gram-staining quality control was performed to check the quality and performance of the reagents by staining Positive controls (colonies of *S. aureus* (ATCC 25923)) and negative controls (colonies of *E. coli* (ATCC 25922)). Media sterility was checked by incubating 5% of the prepared media (batch) overnight and looking for growth after 24hrs. Gram-positive, Gram-negative bacteria, and fungi species were used to check the quality of growing media. The quality of Muller Hilton Agar was checked using *Enterococcus faecalis* isolates and a corresponding trimethoprim-sulfamethazine antimicrobial disk in accordance with CLSI standards. These quality control aspects are included in the revised manuscript.

20. Line 30- Questionnaire a section for questionnaire design and administration in the materials and methods preferably before specimen collection section. Delete everything on questionnaire on this pg and pg 5 and move to the new section for the questionnaire design and administration. How was the question design and based on what? How did you structure your questionnaire? Summary of the questions asked in each section and number of questions? How did you pretest?

***Author’s Response:***

- Comment accepted and these issues are addressed accordingly in the “data and specimen collection” section.

21. Statistical analysis:

This is not detailed enough for the statistical analyses methods tools used for your data. Your descriptive statistics was computed as what? What types of data were collected for this study? Provide comprehensive illustration of steps undertaken for your binary and multivariable logistic regression? What were the independent variables tested for association? What is your dependent? How did you deal with confounding factors? Stepwise illustration of your multivariable model analysis is required? What informed factors (from the univariable analysis) fitted into the model?

***Author’s Response:***

- Comment accepted and these issues are addressed accordingly in the “statistical analysis” section.

22. Results:

Pg 6 Line 8 – delete “around”

***Author’s Response:***

- Thank you and the word “around” was deleted.

23. Line 11- include factors (with p values, COR, and 95% CI) that were associated with UTI at bivariate level before your results for the multivariate regression.

***Author’s Response:***

- Thank you and factors associated with URI at bivariate level are included in the revised manuscript.

24. Pg 7- Line 3 Antimicrobial susceptibility pattern of bacterial uropathogens:

Can you mention these bacteria and their AMR pattern/profile?

***Author’s Response:***

- Thank you for the comment. A detailed antimicrobial susceptibility pattern of each bacterial isolates is provided in Tables 4 and 5. We put minimal information in the text here in order to avoid reptation and redundancy of information. However, we accept the comment and incorporate detail information.

25. Multiple drug resistance patterns of the isolates:

This whole section is not detailed enough and too vague.

Around 85.7% of Gram negative 5 and 89.6% of gram-positive bacteria showed multidrug resistance for the tested antimicrobial 6 drugs (which gram negative bacteria; which gram positive bacteria; to which AMs drugs)

***Author’s Response:***

- Thank you and detailed explanations are provided in the revised manuscript.

26. Discussion:

First paragraph- please can you rephrase? There are some grammatical errors making your thoughts difficult to follow. Please, you will need to rewrite this section. A few of your arguments are really not logical. There are grammatical and typo errors making this hard to follow.

***Author’s Response:***

- Thanks for the comment and grammatical and typo errors are corrected as per the comment in the revised manuscript.

27. Conclusion:

Where participants being managed for UTI?

***Author’s Response:***

- Participants with significant asymptomatic UTI were linked to the nearby public hospital for appropriate treatment.

28. Figure is not clear. I feel this should be deleted, looks repetitive since you have mentioned in the text (the result section).

***Author’s Response:***

- We accept the comment and will not include Figure 1 in the revised manuscript.

**Reviewer #3 comments**

1. The line numbering was not consistent across the body of the manuscript text. Authors need to ensure proper line numbering.

***Author’s Response:***

- Thank you for the comment and we have included proper line numbering across the manuscript.

2. Abstract:

Objectives: The objectives are captured as if the study is yet to be done. Authors need to frame the objectives in past tense.

***Author’s Response:***

- Thanks for your concern, but I think the objective of the study, in the previous version of manuscript, was written in past tense.

3. Methods: p value supposed to be <0.05 and not ≤ 0.05

***Author’s Response:***

- Thank you for your suggestion and we have taken correction.

4. Result: Authors should maintain 1 decimal place for the proportion, and 2 decimal places for the Adjusted odds ratio and 95% confidence interval. P-value should also be in 2 decimal places.

***Author’s Response:***

- Thank you for your positive comment and suggestion. We have made the necessarily change.

5. Materials and methods:

Line number 15: Suggested sequence to follow as follows: Study area, Study design, Study population, Eligibility criteria (Inclusion and Exclusion criteria), Sample size determination, Sampling technique, Data collection method(s) and Data analysis.

***Author’s Response:***

- Thank you and we followed the suggested sequence of sub-sections.

6. Line numbers 17-25: Need for authors to reframe the methods section

***Author’s Response:***

- Comment accepted and modifications are made based on the comment.

7. Line numbers 19-22: Authors need to provide detailed information on how the study areas were selected.

***Author’s Response:***

- We have selected the study areas (4 colleges) out of the total 10 colleges in the town using simple random sampling technique, and we state this, including the participant sampling technique, in the revised manuscript.

8. Line number 23: This is the sample size, authors need to provide detailed information on how the sample size calculation was done.

***Author’s Response:***

- Thank you for the comment and we have provided detailed information regarding sample size calculation in the newly added “sample size determination and sampling technique” section.

9. Line number 22: ‘’female college students’’: These are study population. There is need for authors to incorporate eligibility criteria (inclusion and exclusion criteria)

***Author’s Response:***

- We accept the comment and detailed eligibility criteria (both inclusion and exclusion criteria) are incorporated in our revised manuscript.

10. Line numbers 23-25: Authors should provide detailed information on the sampling technique used to select eligible respondents for interview.

***Author’s Response:***

- Thank you and we have provided detailed information regarding the sampling technique we used to select the study participants.

11. Line number 27: Authors to state the method of questionnaire administration: self or interviewer administered?

***Author’s Response:***

- Thanks for the comment. We have used self-administered questionnaires. This is because the study participants may not be comfortable to respond for some questions while using an interview-based questionnaire. And we mention this in the revised manuscript.

12. Line number 28: Clinical and risk factors data on ……? Need for the authors to provide the need information.

Line number 5 under specimen transportation: ‘’status’’ to be added after non-pregnancy

Line 22 under Cultivation and identification of isolates: was changed to ‘’were’’

Line 28 under Cultivation and identification of isolates: sub cultured changed to sub cultured

Lines 13-20 under Antimicrobial Susceptibility: Drug names to start with Capital letter

Line 25 under Antimicrobial Susceptibility: discs ‘’was’’ measured ……. Change ‘’was’’ to ‘’were’’

Line 26 under Antimicrobial Susceptibility: susceptibility tests ‘’was’’ based …… Change ‘’was’’ to ‘’were’’

***Author’s Response:***

- Comments well taken and the manuscript corrected accordingly, thanks.

13. Line 3 under Quality assurance: questionnaires ‘’was’’ checked by … Change ‘’was’’ to ‘’were’’

***Author’s Response:***

- Thanks for the valuable comment. We accept the comment and corrections are made accordingly.

14. Lines 3-5: ‘’The questionnaire was pretested on 5%..........., Northeast Ethiopia. It will be good if the authors could provide information on feedback from the pre-test and it helped on improving the quality of the study.

***Author’s Response:***

- Thanks for the comment. We have done pretest on 5% of the total sample size (22 asymptomatic female college students) from Mankul college which is located in Dessie city, Northeast Ethiopia. Some modifications were made to the questionnaire after the pretest, particularly on improving the clarity of questions. We have provided this information in the revised manuscript.

15. Lines 21-22: ‘’To show the relationship between each variable……was utilized’’. Test statistic to assess association between dependent and independent variables is Odds ratio (Crude OR) (with 95% CI) OR Chi square (with p-value). This is bivariate analysis. Binary logistic regression is a multivariate analysis used to assess predictors of a dependent or outcome variable. Authors should also provide information on Dependent (outcome) and Independent (explanatory) variables. The sequence supposed to be univariate, bivariate, and multivariate analysis.

***Author’s Response:***

- Comment addressed in the method section, thanks. We followed the sequence to analyze the collected data, and incorporate details to the statistical analysis section.

16. Line 25: Ethical approval and consent to participate: For journal publication, authors should situate the ethical considerations sub-section based on the journal's guidelines.

***Author’s Response:***

- Thank you and we have written a detailed ethical considerations sub-section based on PLOS ONE guidelines.

17. Results:

Line 5: Authors should include the response rate based on the number of eligible respondents reached.

***Author’s Response:***

- We have included all 422 study participants with 100% response rate.

18. Line 9: Table 1 to be included after students

***Author’s Response:***

- Thank you, and we placed ‘Table 1’ at the end of the paragraph according to the comment.

19. Line number 14 ‘’15 independent variables’’; Authors should provide information on the independent variables under the methods section.

***Author’s Response:***

- Thank you, and detailed information on the independent variables are provided in the methods section.

20. Authors should maintain 2 decimal points for information on results section.

***Author’s Response:***

- Thank you, and comment addressed in the results section.

21. Line 16: AOR: Adjusted Odds Ratio. This should be mentioned under the methods section and written in full with the binary logistic regression done.

***Author’s Response:***

- Comment addressed, thanks.

22. Line 20: Authors should use greater than or equal to sign.

***Author’s Response:***

- Comment addressed, thanks.

23. Line 21: (47.9) and (13.8), Authors to insert % to 47.9 and 13.8

***Author’s Response:***

- Comment addressed, thanks.

24. Line 3 under Multiple drug resistance patterns of the isolates: This should be defined under the methods section. Need for authors to include ''Operational definitions'' under the methods section.

***Author’s Response:***

- Thank you. We have added “Operational definitions” sub-section under the methods section and we put the standard definition of multiple drug resistance.

25. Discussion:

The first paragraph of the Discussion should first provide information on summary of the study based on the study objectives. This is important to provide information if the study objectives is achieved or not.

***Author’s Response:***

- Comment addressed, thanks.

26. Discussion:

The first paragraph of the Discussion should first provide information on summary of the study based on the study objectives. This is important to provide information if the study objectives is achieved or not.

***Author’s Response:***

- Thanks for the vivid comment. We have provided a summarized information of the study based on the achievement of study objectives.

27. Line 11 and 18: The use of the ''i.e'' acronym should be deleted.

***Author’s Response:***

- Comment addressed, thanks.

28. Lines 15-16: ‘’This is in agreement with studies in Nigeria’’ This is incomplete. Need for authors to provide information on the type of study conducted.

Line 17: relatively higher than the findings in Ghana: Need to elaborate on the study information in Ghana.

Line 20: ‘’findings was lower….from Nigeria’’: Authors should be explicit on the type of information in relation to Nigeria.

***Author’s Response:***

- Comments addressed in the discussion section, thanks.

29. Line 24 and 25: ‘’Furthermore, bacteriuria among pediatircs peak during ……………….’’ Reference to be provided.

***Author’s Response:***

- Thank you and reference is cited based on the comment.

30. Line 20-23: ‘’Our finding revealed that the frequency of sexual intercourse………: This is repetition of results, authors are advised to delete.

Lines 26-28: ‘’According to our investigation, prevalence of UTI……..’’ This is repetition of results, authors are advised to delete.

***Author’s Response:***

- Comments addressed in the discussion section, thanks.

31. Line 8-11: ‘’ In the present study, there was no statistically significant association between prevalence of UTI in asymptomatic female college students and age, residence (colleges), student batch, monthly family income, marital status, history of UTI, history of catheterization, history of STDs, and presence of genitourinary abnormalities. Similar finding was reported from Nigeria’’. I do not think this statement is important.

***Author’s Response:***

- Thank you for the valuable comment and we have removed the statement accordingly.

32. Line 1: According to the international standard for definition of drug resistance ‘‘This information supposed to be part of the operational definitions under the methods section’’.

***Author’s Response:***

- We provide operational definition in the methods section, thanks.

33. Limitations

Lines 22 and 23: Study limitations are factors which could affect the outcome of the study but however, measures were taken in addressing or mitigating such. The information provided by the authors is not a study limitation.

***Author’s Response:***

- Sorry, we have misinterpreted ‘study limitations’ section. And in this sense, we decide to remove it.

34. Line 24: List of abbreviations: Authors should ensure the list of abbreviations should be captured the way they appear in the body of the manuscript.

***Author’s Response:***

- Thank you for the comment and we address the concern.

35. Figure 1: The title should be underneath the chart.

***Author’s Response:***

- Thank you for the comment. However, we have deleted Figure 1 based on the comment from the other reviewer as information from the figure were also repeatedly provided in the text.

36. Table 1: Unequal class width for respondents age. Authors should ensure class width are equal. What does ETB means? This supposed to be captured under the abbreviation section.

***Author’s Response:***

- Thank you for the comment. We have used age group with equal class width; and we also indicate the full form of ETB in the abbreviation section.

37. Others: under genital area washing habit include the females who wash their vaginal area 5-7 times per day. SUP= Significant Uropathogen, AOR = adjusted odds ratio, COR = crude odds ratio, 1 = reference group, 95% CI = 95% confidence interval, DM = Diabetic mellitus, UTI = urinary tract infection, NA = not applicable. ‘’This is to be included in the abbreviation section.

***Author’s Response:***

- Thank you for the comment. We have included major abbreviations in the list of abbreviations section.

38. Table 3: Frequency of bacterial and fungal uropathogens of asymptomatic UTI among female college students in Dessie town, Northeast Ethiopia, January 2021 - March 2021.: Authors to include N=104 with the title. ‘’from 104’’ to be deleted from N (%).

***Author’s Response:***

- Thank you for the comment. We have included major abbreviations in the list of abbreviations section.

39. Table 3: Frequency of bacterial and fungal uropathogens of asymptomatic UTI among female college students in Dessie town, Northeast Ethiopia, January 2021 - March 2021.: Authors to include N=104 with the title. ‘’from 104’’ to be deleted from N (%)

***Author’s Response:***

- Comment addressed in Table 3, thanks.

40. Antimicrobial resistance pattern (R0-R5) in Table 6: The antimicrobial resistance pattern supposed to be mentioned under the operational definitions of the methods section.

***Author’s Response:***

- We have already included MDR definition in the ‘operational definitions’ section, thanks.

Finally, we thank you for your critical review of the paper.

Sincerely,

Berhanu Kebede Reda, MSc
