# Supplementary material for: Antibiogram of uropathogens and associated risk factors among asymptomatic female college students in Dessie town, Northeast Ethiopia
Source: PLoS One. 2023 Nov 29;18(11):e0276033. doi: 10.1371/journal.pone.0276033 (PMC10686425; doi:10.1371/journal.pone.0276033)
Supplement: Attachment — Submitted filename: Review.docx [file pone.0276033.s006.docx]

**Title: Antibiogram of uropathogens and associated risk factors among asymptomatic female college students in Dessie town, Northeast Ethiopia**

**Manuscript Number PONE-D-22-26752**

I acknowledge the editor of PLOS ONE for inviting me to review this manuscript. I would also like to appreciate the authors for doing this research.

**General comments:**

The manuscript has been well written but still has errors and needs an improvement. There are grammatical and punctuation issues that need revision.

Abstract:

Line 13: “Ten mL”, need capitalization issue

Line 24: 24 (92.3), need and edition by adding “%”

Introduction

Line 2-6: is a full of grammatical errors and scientific justification is required. The expression “Asymptomatic uropathogens” should be revised and rewritten in the right way because infected parson, not the uropathogen will develop symptom or remain asymptomatic.

“Asymptomatic uropathogens” occur frequently and are “a” major cause of UTI leading to unnecessary treatments 5 [2]). It affects millions of people globally with possible conversion into severe symptomatic infections, “especially in developed countries where the healthcare coverage is comparably low.”

Page 5:

Line 11-21, need rearrangement and merging of related texts and put numerical data in different studies in ranges. But the unrelated groups”HIV” patients” should better be removed.

Line 26-29: the sentence is long complex with grammatical errors.

MATERIAL AND METHODS:

“Memhran College (MC)”

Page 7

Line 6: How could the variable “educational level” be important? All study participants and were college students.

Line 17-19 “…within two hours for processing after confirming their non-pregnancy status by using 19 human chorionic gonadotropin (HCG) card pregnancy test.” How much was the period of delay before HCG testing?

Line 24: “Cultures were incubated overnight” dear authors please revise this and replace “cultures” by inoculated media or …media or plates.

Results

Page 10: The median age of the study participants was 21 years, ranges from 18 – 38 years

Is 18–38 the interquartile range? Is 18 years the lowest range?

Page 11:

Line 4-5: “Majority” of the study 5 participants 209 (49.4%) were in the age group of 21 to 25 years. how less than half (49.4%) can be majority?

209 (49.4%)

Line 26-28: Probably the strength of this study is the sample size“…, this study took a small number of asymptomatic female students …”

Discussion:

Page 14:

Line 11-16: “Unlike most of the studies reported in our country and elsewhere in the world, our findings showed majority of the etiological agents for Candiduria were C. troipcalis (48.9%), followed by C. albicans (21.2%).”

Would the authors discuss adequately on why shift from C. albicans to C. troipcalis was seen?

Page 15:

Line 5-6: Age (“17”-30 years) are sexually active with multi-sexual partners? Which age group was your population?

Which age “15, 17 or 18” years the lowest range. On page 10: line 3-4, 18 it seems to be 18 years, Page 15, Line 5-6: “17” and in the table, it is mentioned as 15-20, …

Dear authors:

Is your research spotless? I haven’t see any limitation?

A high rate of MDR has been reported but there is no recommendation regarding this serious issue. There has to be recommendation about MDR (87.7%) infection.
