# Supplementary material for: Antibiogram of uropathogens and associated risk factors among asymptomatic female college students in Dessie town, Northeast Ethiopia
Source: PLoS One. 2023 Nov 29;18(11):e0276033. doi: 10.1371/journal.pone.0276033 (PMC10686425; doi:10.1371/journal.pone.0276033)
Supplement: Attachment — Submitted filename: Response to Reviewers.docx [file pone.0276033.s007.docx]

**Author’s Response to Editorial and Reviewer comments**

**Manuscript ID number:**
PONE-D-22-26752R2

**Title of paper:**
Antibiogram of uropathogens and associated risk factors among asymptomatic female college students in Dessie town, Northeast Ethiopia

**General**

First of all, we acknowledge PLOS ONE for giving us a second chance to revise our manuscript. We also thank all the reviewers for critically reviewing our manuscript which helps us to render better clarity to the paper and make it scientifically plausible. We thank the PLOS ONE academic editors for their valuable comments and for giving us an opportunity to revise the manuscript.

All the questions raised by the Reviewers and Editor have been addressed; and the manuscript is modified accordingly. Moreover, all the requested Editorial corrections are addressed in both the revised manuscript and the response letter. Changes are shown with track changes in the file labeled ‘Revised Manuscript with Track Changes’. The point-by-point response is given below.

**Journal requirements:**

1. Please review your reference list to ensure that it is complete and correct. If you have cited papers that have been retracted, please include the rationale for doing so in the manuscript text, or remove these references and replace them with relevant current references.

***Author’s Response:***

- Thanks for the significant editorial comment. We certainly ensure that our list of references is complete and correct. Moreover, we did not cite any paper that has been retracted.

**Reviewer #2 comments**

1. Abstract: Background: Line 3 - You need to open and close the bracket - (on asymptomatic bacteriuria and asymptomatic candiduria)

***Author’s Response:***

- Thanks for your comment and we open and close brackets on the specified location based on the comment.

2. Methods: Line 13 – Ten mLs.

***Author’s Response:***

- Thank you for the substantial comment. Up on the provided comment, we modify the mL unit to mLs.

3. Introduction: Lines 27-29- provide reference

***Author’s Response:***

- Comment well taken and references are cited for the statement from lines 27 to 29.

4. Material and methods: Talk about the study area before the design.

***Author’s Response:***

- Thank you for the valuable comment. We correct the statement based on the given comment. As a result, study area and period were mentioned before the study design.

5. Antimicrobial susceptibility testing: Line 2- - change to 5mLs

***Author’s Response:***

- Thank you and corrections are made and the unit is changed to 5 mLs.

6. Quality assurance: Line 12-rephrase to read “An hour training was given to data collectors on data collection”.

***Author’s Response:***

- Comment accepted and the statement is rephrased accordingly.

7. Quality assurance: Lines 12-16 looks like a repetition “The questionnaire was pretested on 5% of the sample size (22 asymptomatic female students) from Mankul College which is located in Dessie city, Northeast Ethiopia, and necessary modifications were made to the data collection tool accordingly, particularly to improve the clarity of questions”

***Author’s Response:***

- Thank you for the valuable comment. We apologize for the repeated statement in the ‘Data and specimen collection’ and ‘Quality assurance’ sections. We noticed the repetition and removed the statement found in the ‘Data and specimen collection’ section.

**Reviewer #4 comments**

1. Abstract: Line 13: “Ten mL”, need capitalization issue. Line 24: 24 (92.3), need and edition by adding “%”.

***Author’s Response:***

- Thanks for your constructive comment and we have made the necessary amendments based on the given comment. We modified as “Ten mLs” and “92.3%”.

2. Introduction: Line 2-6: is a full of grammatical errors and scientific justification is required. The expression “Asymptomatic uropathogens” should be revised and rewritten in the right way because infected parson, not the uropathogen will develop symptom or remain asymptomatic.

“Asymptomatic uropathogens” occur frequently and are “a” major cause of UTI leading to unnecessary treatments [2]). It affects millions of people globally with possible conversion into severe symptomatic infections, “especially in developed countries where the healthcare coverage is comparably low.”

***Author’s Response:***

- Thanks for your constructive comment and suggestion. Based on your comment, we have made improvements to the grammatical errors and modified the whole paragraph accordingly.

3. Page 5: Line 11-21, need rearrangement and merging of related texts and put numerical data in different studies in ranges.

***Author’s Response:***

- Thank you for the comment. We have rearranged and merged texts in the paragraph and put the numerical data of different studies in ranges. We modified the paragraph as follows: “The majority of studies conducted in Ethiopia have primarily focused on pregnant women, revealing a range of prevalence rates for bacteriuria (8.5% to 18.8%) across various regions of the country [20-26].”

4. Line 26-29: the sentence is long complex with grammatical errors.

***Author’s Response:***

- Thank you for your helpful comment, and the complex and long sentence is now modified and grammatical errors are corrected.

5. Material and methods: “Memhran College (MC)”

***Author’s Response:***

- We thank the reviewer for the detailed observation. And we corrected the non-uniformity of the spelling for “Memhran College (MC)”. It is now corrected to “Memheran College (MC)” and modifications are made accordingly.

6. Page 7: Line 6: How could the variable “educational level” be important? All study participants were college students.

***Author’s Response:***

- Thank you for your hands-on comment. And we apologize for using ‘educational level’ as a variable. But we meant to use ‘student batch’ as in 1^st^, 2^nd^, 3^rd^ and 4^th^ year. Thus, we have modified it into “student batch”.

7. Line 17-19 “…within two hours for processing after confirming their non-pregnancy status by using 19 human chorionic gonadotropin (HCG) card pregnancy test.” How much was the period of delay before HCG testing?

***Author’s Response:***

- Comment well taken and statement is rephrased. HCG pregnancy test is done immediately after collection, and we have incorporated this in the revision.

8. Line 24: “Cultures were incubated overnight” dear authors please revise this and replace “cultures” by inoculated media or …media or plates.

***Author’s Response:***

- Thank you for your constructive comment. We totally accept the comment and replace “Culture” by “Inoculated plates”.

9. Results:

Page 10: The median age of the study participants was 21 years, ranges from 18 – 38 years. Is 18–38 the interquartile range? Is 18 years the lowest range?

***Author’s Response:***

- Comment accepted and amendments are made. 18 – 38 years is the age range of participants (18 the lowest and 38 the highest). The median and interquartile range (IQR) is 21 years (IQR 20 to 23 years). We have modified the statements as follows: “The age of study participants ranged from 18 to 38 years, with a median age of 21 years, and an interquartile range (IQR) of 20 to 23 years.”

10. Line 4-5: “Majority” of the study 5 participants 209 (49.4%) were in the age group of 21 to 25 years. how less than half (49.4%) can be majority? 209 (49.4%).

***Author’s Response:***

- Thank you for the valuable input. We meant to say majority from the 4 age groups i.e., 172 (40.7%), 209 (49.4%), 25 (5.9%) and 16 (3.8%). We indeed accept the comment and modifications are made accordingly. The statement is corrected as “About half of the study participants 209 (49.4%) were in the age group of 21 to 25 years.”

11. Line 26-28: Probably the strength of this study is the sample size“…, this study took a small number of asymptomatic female students …”

***Author’s Response:***

- The study indeed took an adequate number of sample size. We included a total of 422 female college students who were asymptomatic for UTI. Thus, all the participants were asymptomatic female college students.

12. Discussion:

Page 14: Line 11-16: “Unlike most of the studies reported in our country and elsewhere in the world, our findings showed majority of the etiological agents for Candiduria were *C. troipcalis* (48.9%), followed by C. albicans (21.2%).”

Would the authors discuss adequately on why shift from *C. albicans* to *C. troipcalis* was seen?

***Author’s Response:***

- Comments taken and detailed scientific justifications were incorporated on why shift from *C. albicans* to *C. troipcalis* was seen.

13. Page 14: Line 5-6: Age (“17”-30 years) are sexually active with multi-sexual partners? Which age group was your population?

Which age “15, 17 or 18” years the lowest range. On page 10: line 3-4, 18 it seems to be 18 years, Page 15, Line 5-6: “17” and in the table, it is mentioned as 15-20, …

***Author’s Response:***

- Thank you and the age group in the specified location (17 – 30 years) is corrected to (18 – 38 years) which is exactly the age range of our study participants, with the lowest range of 18 years and an upper range of 38 years. However, in Table 1, we have 15-20, 21-25, 26-30 and >30 years which are just age categories. To further create no ambiguity and based on the given comment, we modified the age group (15-20) to ≤20.

14. Dear authors: Is your research spotless? I haven’t see any limitation?

***Author’s Response:***

- Comments taken and limitation and strength of the study are provided at the end of the discussion section accordingly, thanks.

15. A high rate of MDR has been reported but there is no recommendation regarding this serious issue. There has to be recommendation about MDR (87.7%) infection.

***Author’s Response:***

- Thank you and a concluding remark regarding the high rate of MDR is now provided at the end of the conclusion section.

Finally, we thank you for your critical review of the paper.

Sincerely,

Berhanu Kebede Reda, MSc
